# The N400 effect captures nuances in implicit political preferences

**DOI:** 10.1038/s41598-024-67763-7

**Published:** 2024-07-20

**Authors:** Emmanuel Mahieux, Lee de-Wit, Leun J. Otten, Joseph T. Devlin, Nicole Y. Y. Wicha

**Affiliations:** 1 Department of Experimental Psychology, University College London, London, UK; 2 Department of Psychology, University of Cambridge, Cambridge, UK; 3 Institute of Cognitive Neuroscience, University College London, London, UK; 4 Department of Neuroscience, Developmental and Regenerative Biology, University of Texas at San Antonio, San Antonio, USA

**Keywords:** Reading, Social behaviour, Human behaviour

## Abstract

We conducted a study in San Antonio, Texas, in the weeks preceding the 2022 state Governor election to determine if implicit or explicit measures of political preference could predict voter behavior. We adapted an established event-related potential (ERP) paradigm showing political statements to participants one word at the time where the last word made the statement pro-Republican or pro-Democratic. Our sample of college students included decided and undecided voters, and was reflective of the demographic make-up of south-central Texas. Our implicit measures were an established authoritarianism scale and the N400 effect to the sentence-final word. The N400 is an ERP to any stimulus that engages semantic memory and has been shown to measure implicit disagreement with political statements. Explicit measures of political preference and authoritarianism were predictive of vote choice. The expected N400 effect was found for Democratic voters, with larger amplitude to pro-Republican than pro-Democratic statements. Surprisingly, decided Republican voters showed no difference in N400 responses to pro-Republican and pro-Democratic statements and there was no group difference in the N400 effect. In turn, the N400 was not predictive of voter behavior. We argue that the N400 effect reflected individual political preferences, but that ultimately voter behavior aligned with partisan identity.

## Introduction

The former Downing Street head of communications Alastair Campbell once commented that polls hide a complex process. Individual voters make up their minds for one party or the other for a myriad of reasons. The political science and psychology literature has explored how factors that voters are themselves aware of might influence their vote, such as ideology^1^ or how much voters say they personally like one candidate over the other^2,3^.

However, research suggests that voters are not always conscious of all the reasons why they vote and that subconscious factors of voting behavior can affect cognition. Subconscious influences on voting behavior can make voters in situations of fear more likely to choose political leaders with facial features associated with trustworthiness^4^. Cognitive dissonance, the psychological need to be consistent in our behavior and beliefs^5,6^, can have a powerful influence on how people vote. Voters who voted for the party in power will perceive the economy as doing better than those who voted for the party in opposition^7,8^, which in turn makes them more likely to vote again for the same party.

Because some factors shaping voting behavior are not conscious, and therefore not explicit in voters’ attitudes and beliefs, measures of implicit preference offer considerable promise in shedding light on voting behavior and its underlying cognitive processes. Event-related potentials (ERPs) may offer a measure of this kind of unconscious processing which is unlikely to be biased by any of the demand characteristics shaping verbal reports or behavioral data. ERPs are scalp-recorded, continuous measures of electrical brain activity that provide a window into the unfolding cognitive processes that lead to a behavior. Galli et al.^9,10^ proposed that the N400, a well-studied ERP component^11^, could be used as a new measure of implicit political preferences.

The N400 is a negative-going voltage deflection that peaks around 400 ms after stimulus onset and is largest over centro-parietal sites with a slight right hemisphere bias for written words. It is part of the normal response to stimuli that will produce some activity in long-term semantic memory^12^. In other words, the N400 is a response to any meaningful or potentially meaningful stimulus and is larger in amplitude when words (or concepts) are inconsistent with expectations based on context or with internal beliefs/knowledge^11,13^. The N400 is an indicator of semantic processing with the size of its effect varying with the degree of semantic deviation. The N400 effect is the difference obtained from subtracting ERPs for two conditions. For example, the ERP generated by expected stimuli subtracted from the ERP generated by unexpected stimuli could be referred to as an N400 effect of expectancy. For example, “I drink my coffee with milk and ***socks***” will generate a greater ERP response at the word “socks” than reading “I drink my coffee with milk and ***sugar***”^11^.

The N400 has also been found to occur in instances where moral or political expectations are violated. In these instances, the N400 has been hypothesized to be a marker of implicit moral and political preferences. Van Berkum et al.^14^ found that strict Christians and non-Christians had opposite N400 responses when viewing statements on societal issues such as euthanasia, abortion and homosexuality. Notably, participants’ N400s were greater when reading statements they disagreed with than when reading statements they agreed with. For example, strict Christians had larger N400 responses when reading “I think euthanasia is an ***acceptable*** course of action”, which is inconsistent with their moral values, than when reading “I think euthanasia is an ***unacceptable*** course of action”. The idea formulated by the authors is that any concept stored in semantic memory has an affective tag attached to it, which will mark it with positive or negative valence when retrieving its meaning.

Galli et al.^9,10^ also found evidence supporting the claim that violations of moral or political expectations elicit the N400. In the weeks preceding the 2016 EU referendum in the UK on whether to leave the European Union or remain in it, Galli and her colleagues compared the N400 to key words in political statements between decided and undecided voters. Consistent with Van Berkum et al.^14^, they observed that decided Leave voters had greater N400 responses when reading pro-Remain statements, while Remain voters had the inverse pattern with greater N400 responses when reading pro-Leave statements. Critically, they found that the N400 effect predicted the vote of both decided and undecided voters above and beyond a measure of explicit preference as measured by a self-report index (SRI).

Galli et al.^10^ reimplemented this paradigm at the 2019 European parliamentary elections in Italy. They examined whether the N400 effects generated by separate political issues, namely economic, anti-establishment and cultural issues, were predictive of voter behavior in mainstream versus populist voters. They also tested how this measure compared with another measure of implicit political preference—the implicit associations test (IAT)^15^—and with an explicit measure of political preference—an SRI. The N400 was larger in amplitude for statements on economic issues that were inconsistent with participants’ political attitudes but was not different between groups for anti-establishment and cultural issues. They also found that the N400 for statements on economic issues was significantly predictive of vote choice as a standalone predictor, although not above and beyond the explicit measure of political preference, while the IAT was not. Moreover, the model that included both the SRI and the N400 effect as predictors was better at predicting vote choice than the model with only the SRI. Their findings suggested that political preferences on economic issues, which have traditionally been useful predictors of voting behavior^16–18^, remained a key factor shaping vote choice.

Morris et al.^19^ used the N400 to garner support for the “hot cognition” hypothesis which posits that all sociopolitical concepts are affectively charged, and that this affective charge is automatically activated from long-term semantic memory within milliseconds of political stimulus presentation. They found that prime and target pairs of political attitude objects elicited greater negativities in the N400 latency range when the prime and the target were affectively incongruent compared to when they were congruent. The authors interpreted these results as suggesting that “the emotional valence of a political prime is stored along with the concept itself, and that an affective response becomes active upon mere exposure to the political stimulus”^19^ (p. 1).

To summarize, we currently know several aspects about the relationship between the N400 and political behavior: (1) The N400 response is greater for political or moral statements that are inconsistent with our beliefs and in this way can act as an implicit measure of disagreement. (2) The difference in the N400 response across political groups aligns with political beliefs, for example with Leave and Remain voters presenting opposite N400 effects to the same political statements. (3) In Galli’s 2019 study, mainstream and populist voters had similar responses to statements on anti-establishment and cultural issues. They differed only in their responses to statements on economic issues. Thus, not all political stimuli generate different N400 responses in different political groups, despite their explicit preferences. (4) Based on Galli’s findings, the N400 effect appears to improve the predictive ability of models with explicit measures of political preference by accounting for some of the variance in voting behavior that these measures do not account for.

Our study aimed to add to this existing knowledge in two ways. First, we tested if the N400 effect was predictive of vote choice in a different, highly polarized electoral context in the United States, using an adaptation of Galli et al.’s N400 paradigm. Should the N400 effect to political statements align with vote choice in decided voters, we can use this implicit measure of political preference to predict undecided voters’ vote choice retrospectively. We sought to compare the ability of these implicit measures and that of explicit measures of political preference in predicting vote choice.

The Texas 2022 gubernatorial election in San Antonio provided an ideal political environment to test this. Texas has been a Republican-led state since 1995 within which the district of San Antonio is a Democratic enclave. The gubernatorial election took place two years after Democratic president Joe Biden’s election to the presidency and less than five months following the Supreme Court’s decision to reverse Roe v Wade, a landmark ruling that had enshrined a woman’s right to abortion since 1973. Moreover, the incumbent Republican governor of Texas, Greg Abbott, who was seeking re-election, had recently signed Senate Bill 8 of the Texas legislature, better known as the “heartbeat bill”, which banned abortions after the first six weeks of pregnancy, making abortion a salient issue in the political debate. The Democratic candidate, Beto O’Rourke, opposed this measure^20^. Moreover, the political climate was marked by an immigration crisis with high numbers of immigrants crossing from Mexico into Texas which the state government blamed the federal government for^21,22^.

Thus, the political climate of San Antonio and Southern Texas in the weeks preceding the election made fault lines between the two parties and their candidates particularly salient. It provided the ideal setting to test whether the N400 effect could reflect the differences in political preferences between Republicans (right leaning) and Democrats (left leaning) similarly to what was observed between Leave and Remain voters in the UK, and populist and mainstream voters in Italy. If the N400 effect is a marker of implicit disagreement, it should reflect political differences between opposing sides and be a strong predictor in a highly polarized political environment.

Consider the statement, *The ownership of automatic assault weapons like AR-15 s needs to be restricted/protected*. Based on Galli et al.’s findings, if voters hold pro-Democrat or pro-Republican views, they should have a larger N400 response when reading the target word that runs contrary to their beliefs. Democratic participants should have more negative-going responses to Republican** (protected)** than Democratic statements (***restricted)***, and vice-versa. We hypothesized that the N400 effect would constitute an implicit measure of political expectations and beliefs at the target word. In turn, our goal was to determine if the N400 effect derived from reading the target words was predictive of vote choice in undecided voters.

Second, in addition to measuring implicit political preferences with the N400 effect, we used another implicit predictor of voting behavior: namely a recently revised measure of authoritarianism by Engelhardt et al.^23^ that focuses on child-rearing values with no explicit reference to politics. Authoritarianism is a personality adaptation that values social cohesion and conformity to in-group norms over personal freedom and individual autonomy^23^. Authoritarianism was used as a factor along with the N400 effect in a model of vote behavior to further test how implicit, non-political measures of political preference compared with explicit measures of political preference in their ability to predict vote choice. Authoritarianism has been a good predictor of voting for Trump and other far-right leaders in the past years^24^. Authoritarianism is also a correlate of several factors that influence political beliefs and voting behavior, such as attitude towards outgroups^25^, race and gender^26^ and political alignment with the far-right^27^. Stenner and Haidt^24^ found that in situations of high normative threat, the probability for voters with high authoritarianism scores of voting for a far-right candidate in the US or France was respectively 87 and 84% compared to 7 and 11% for voters with low authoritarianism scores.

We examined whether authoritarianism could also explain divides in our sample of the US electorate. The ability of measures of extreme-left attitudes to predict the kind of voting behavior we are interested in has not been tested, to our knowledge, contrary to authoritarianism, which motivates our choice to use the latter but not the former. We used two measures of authoritarianism: Engelhardt et al.’s^23^ child-rearing values scale and Bizumic’s^28^ very short authoritarianism scale (VSAS). Engelhardt et al. developed a measure of authoritarianism that constitutes an implicit measure of political preferences. It relies on asking participants which values they consider most important in raising children, for example if it is more desirable for a child to be curious or to have good manners. It is considered an “implicit” measure of political preferences because it measures an attitude (authoritarianism) that underlies political preferences rather than an explicit agreement or disagreement with political preferences.

The advantage of using the child-rearing values scale is that it reliably measures authoritarian attitudes and their political correlates without using wordings that are explicitly political, thus avoiding the risk of the dependent and independent variables being indistinguishable and allowing us to test the predictive potential of implicit authoritarian attitudes. This risk of the dependent and independent variables being endogenous can occur when predictors attempt to explain political attitudes and behaviors (e.g. vote choice, policy preferences) with other political attitudes and behaviors (e.g. self-placement on a left–right scale, right-wing authoritarianism scale) which constitute the same phenomenon.

In addition to the implicit child-rearing scale by Engelhardt et al., we included an explicit measure of authoritarianism in order to compare their respective abilities to predict vote choice. We thus included Bizumic’s VSAS which uses Likert ratings to measure authoritarianism with explicitly political statements, such as “What our country needs most is discipline, with everyone following our leaders in unity”.

We tested whether our implicit measures of the N400 effect and child-rearing values scale predicted vote choice. We predicted that the N400 effect would reflect the differences in explicit political preferences between decided Republicans and decided Democrats in the same way that it reflected differences between voters in the UK and Italy. The N400 effect should align with political preferences, with larger N400 amplitude for target words that are inconsistent with political preferences compared to target words that are consistent with them. Thus, we predicted that Democrats should have more negative-going N400 amplitudes for pro-Republican compared to pro-Democratic target words whereas Republicans should have more negative-going N400 amplitudes for pro-Democratic compared to pro-Republican target words. We further tested this hypothesis for different political issues—the economy, immigration and societal issues- to see if we could replicate Galli et al.’s^10^ findings on political-issue related N400s. We hypothesized that if the N400 effect aligned with vote choice in decided voters, it would predict undecided voters’ vote choice above and beyond explicit measures, in line with Galli et al.’s 2017 findings^9^. We also predicted that our implicit measure of authoritarianism (the child-rearing scale) would be a better predictor of voting choice among all voters than the explicit measure of authoritarianism (the VSAS).

The current study replicates and extends the N400 studies conducted in Europe to a young adult population in the US during a highly polarized political period. By using a multi-measure approach (e.g. neural, behavioural and survey data), together these findings contribute to the neuro-politics literature by examining how different implicit and explicit measures of political preference impact vote choice.

## Methods

### Experimental procedure

#### Participants

The study took place at the University of Texas at San Antonio (UTSA) in the weeks preceding the 2022 Texas gubernatorial election, from 14th October to 10th November 2022. The study received IRB approval for research with human participants (protocol FY21-22-345) from UTSA’s Institutional Review Board. All experiments were conducted in accordance with IRB guidelines and regulations and informed consent was obtained from all participants.

We recruited 55 participants among undergraduate students registered to vote and planning to do so (Female = 32, Male = 22, Non-binary = 1). UTSA students’ racial and ethnic backgrounds (Hispanic or Latino = 59%, White = 21%, Black or African American = 8%, Other = 12%^29^) reflect the proportions in the general South-central Texas population. The average age was 20 years (SD = 2.4 years). All participants were right-handed, with normal or corrected to normal vision. Among the participants, 20 had decided to vote for the Democratic candidate for state governor, 18 for the Republican candidate and 17 were undecided as to who to vote for.

Voting intention was used in the pre-screening to determine eligibility for the study. Vote intention was coded as a Likert scale with Democrat = 1 and Republican = 5 and had five possible answers: Democrat, Leaning Democrat but not sure, Undecided, Leaning Republican but not sure and Republican. Although Texas effectively is a two-party system, there are other party affiliations such as the Libertarian and Green parties^30^. The Libertarian party promotes a political vision based on individual liberty which has endorsed both economically conservative and, on certain issues, socially liberal policies such as opposing the imposition of income tax and supporting gay marriage. Participants who intended to vote for another party than the Republican or Democratic party were not eligible for the study as the study paradigm could only predict binary outcomes. We also collected partisan identity data and voting history. Over 75% of participants (42 out of 55) were first-time voters so voting history could not be used effectively.

We recorded participants’ gender and race/ethnicity. Participants were asked to select all ethnic categories that applied, leading to 9 different factorial levels. We chose to include race because it is typically used in US elections to characterize different electorates.

Of the 55 participants in our study, 31 cast a vote for the Democratic candidate for governor and 24 for the Republican candidate. EEG and behavioral data were collected prior to participants casting their ballot, starting 25 days before the vote and up to the day of the vote itself (November 8th). Five decided Republican voters’ data were collected after they cast their ballot with the goal of having equally sized samples across groups. These additional data were collected after they had cast their vote because we ran out of time to collect data from decided Republicans before the election and it was essential to have comparable sample sizes among decided Republicans and decided Democrats. However, the fact that they had already voted did not affect the validity of their data because their N400 effects were used to validate the link between the N400 effect and their explicit preferences rather than to predict their vote.

Among Democratic voters, 20 were decided at the time of the experiment on who to vote for before the election while 11 were undecided. Among Republican voters, 18 were decided and 6 undecided. Participants’ vote choice for Texas governor was obtained by following up with participants by email or phone in the days following the November 8th election.

#### Predictors

We aimed to determine whether the N400 effect is a significant predictor of voting behavior and how it compares to other predictors. For this reason, we tested the ability of different neural, political, behavioral and demographic variables in predicting participants’ vote for governor. We inserted each of these variables as the single predictor of a logistic regression predicting vote choice. We describe each of these predictors in turn.

Implicit measures of political preference included authoritarianism as measured by the child-rearing values scale, the overall N400 effect and the N400 effects for each issue dimension (economy, immigration, societal). Explicit measures included the VSAS, perception of the economy, the president’s job approval, the most important problem facing the country, political knowledge and an Explicit (political) Preference Index (EPI). We explain the N400 effect and EPI measures together with the ERP experimental materials below.

The **child-rearing values score**^23^ constitutes a measure of authoritarianism. The child-rearing values scale was assembled by showing respondents pairs of values which are desirable for children to be raised with. Respondents were asked to say which value in each pair they thought was more important for a child to have. These pairs are:

- Independent or respectful of their elders?
- Curious or to have good manners?
- Obedient or self-reliant?
- Considerate or well-behaved?
- Free-spirited or polite?
- Orderly or imaginative?
- Adaptable or disciplined?
- Loyal or open-minded?

A participant’s score was computed by subtracting the number of authoritarian responses from the number of non-authoritarian responses. The advantage of the child-rearing values scale is that it is a proven proxy of authoritarianism despite being exogenous to political attitudes.

The **very short authoritarianism score (VSAS)**^28^**,** in contrast to the child-rearing values score, is an explicit measure of an individual’s propensity to authoritarianism. It captures the different facets of authoritarianism that have traditionally been measured with the longer Right-Wing Authoritarianism scale^31^ but does so with a reduced set of statements. It was assembled by asking respondents the extent to which they agreed or disagreed with the following statements:

- It’s great that many young people today are prepared to defy authority.
- What our country needs most is discipline, with everyone following our leaders in unity.
- God’s laws about abortion, pornography, and marriage must be strictly followed before it is too late.
- There is nothing wrong with premarital sexual intercourse.
- Our society does NOT need tougher government and stricter laws.
- The facts on crime and the recent public disorders show we have to crack down harder on troublemakers, if we are going to preserve law and order.

**Economy perception**. We asked participants “thinking about the nation’s economy, how would you rate economic conditions in the country today?”, using the wording of the Pew Research Institute. Perceptions of how the economy is doing have long been a bellwether for voting intentions^16–18^, with the assumption that voters tend to hold the incumbent president or/and the party in power responsible for the state of the economy.

**President's job approval**. We asked participants to indicate whether they approved or disapproved of Joe Biden’s job as president.

**The most important problem facing the country**. Polling agencies ask respondents to indicate what for them is the most important issue facing the country. Responses to such surveys are used to analyze public opinion ahead of elections^32^. We borrowed the wording of this question from the political polling and analytics company Gallup that has well-established methodologies for analyzing public opinion:

Which of the three issues below do you think is the most important problem facing the country today?

- The Economy
- Immigration
- Societal issues (abortion, race relations, gun rights)

**Political knowledge** was measured with a questionnaire^33^ designed by the Pew Research Centre.

Our dependent variable was participants’ **vote choice for Texas state governor**. This factor variable had two levels: Democrat or Republican. Data for this variable were collected after participants had effectively cast their vote.

#### Materials

The stimuli included 184 statements that were each 11 words in length. A minority of these words included contractions, apostrophes or hyphens (e.g. “Abbott’s”, “covid-19”). Stimuli consisted of political statements on the issues most important to Texan voters. These were determined on the basis of the issue tracker of the University of Texas at Austin’s Texas Politics Project (https://texaspolitics.utexas.edu/) which indicated three political issue dimensions as being the most important for Texas voters: the economy, immigration and societal issues (i.e., gun rights, abortion, Black Lives Matter, fairness of elections). Certain statements did not fall in any of these issue dimensions and were included in a general issue category which counted towards the overall N400 effect but not towards issue-specific N400 effects.

Each statement ended with a target word that made the statement pro-Democrat or pro-Republican. There were thus two versions of 92 statements for a total of 184 statements which are included in the Supplementary Information. All participants saw both versions of all statements. There were 46 statements pertaining to the Economy, 36 statements pertaining to Immigration and 56 statements pertaining to Societal issues. The uneven number of statements for each issue dimension was mainly due to the fact that issues like the economy and societal issue are broader than immigration and allowed for a greater number of statements to be generated. The statements were divided into two blocks (A and B), with each block containing only one version of each statement. Block B had the opposite endings than block A.

In order to avoid order effects, statements were pseudo-randomised and block order was alternated across participants. This was done by randomizing statement order and adjusting it so that there would not be more than 2 consecutive Democratic or Republican statements throughout the experiment. We subsequently ran an ANOVA with block order as between-subject factor to check whether there was any difference in N400 amplitude between participants who were shown A and then B versus those who were shown B then A. The main effect of block order was not significant, indicating that N400 amplitude did not differ according to block order.

Participants were shown 10 practice trials (the same for all participants) to familiarize them with the task. A full list of the 184 statements used is included in the supplementary information. Examples of the statements include:

- Immigration: We see an immigration crisis because of the policies of ***Biden/Abbott***.
- Societal: The decision to interrupt a pregnancy is something up to ***God/women***.
- Economy: The Democrats’ economic agenda will lead the US economy to ***growth/recession***.
- General issue: The people who stormed the Capitol on January 6th were ***patriots/thugs***.

The wordings of the statements were adapted from statements of the 2020 American National Election Study User Guide and Codebook^34^ and from declarations of the Democratic and Republican candidates for governor so that they would be identifiable as pro-Republican or pro-Democrat. To ensure that statements reflected identifiable partisan views, they were pre-tested by a random sample of 15 Democratic and 15 Republican Texas voters recruited on Prolific who were asked to indicate whether each stimulus reflected the views of the (1) Republican party, (2) the Democratic party, (3) neither of the two parties, (4) both parties or if they (5) didn’t know. Only statements that were indicated by over 70% of testers as reflecting the views of the Republican or Democratic party were included in the study.

**Explicit preference behavior**. Participants assessed each statement through a button-press to state whether they agreed or disagreed with each statement. The **explicit preferences index** (EPI) constituted an explicit measure of political preferences and was measured during the ERP experiment when participants explicitly stated whether they agreed or disagreed with each political statement. We borrowed the methodology for calculating the EPI from the Kelley index^35^, to quantify the degree of agreement of each participant with the Democratic or the Republican party platform. It is calculated in the following way:

EPI = (% Republican statements agreed + % Democratic statements disagreed) - (% Republican statements disagreed + % Democratic statements agreed).

Similar to the N400 effect, more positive values indicate greater agreement with Republican views while more negative values indicate greater agreement with Democratic views. A score of 200 would indicate perfect alignment with the Republican party while a score of − 200 would indicate perfect alignment with the Democratic party.

#### Procedure

Upon arrival, participants were asked to fill in a consent form and screened for covid-19 symptoms. They also completed a questionnaire on their educational attainment, age and socio-demographic background before or after the EEG session. They completed a questionnaire on their voting history, political knowledge, party affiliation, voting intentions, child-rearing values, authoritarianism score, perception of the state of the economy and their approval of the current president before the EEG session.

Participants were fitted with an EEG cap and sat in front of a computer screen on which the stimuli were displayed. Ten practice trials preceded the main experiment and were not repeated afterwards. Words in black font were shown one at the time at the center of a white screen using Paradigm, a stimulus presentation software with an EEG integration (http://www.paradigmexperiments.com). The lights inside the recording chamber were dimmed. Trials began with a “ + ” sign fixation mark for 1000 ms, and then each word appeared for 200 ms, followed by a 300 ms blank screen. This rapid serial visual presentation allowed participants to read with as little eye movement as possible to prevent ocular artifacts from contaminating the EEG signal of interest.

The final target word was presented for 1000 ms, followed by a screen asking participants if they agreed or disagreed with the statement. When participants made their agree or disagree response, the sentence-final word was no longer on the screen. To move to the next screen, participants responded with a button press on a Playstation joystick. The response button that indicated agreement or disagreement was counterbalanced across participants.

#### EEG recording and pre-processing

EEG was acquired with a 26-electrode setup in geodesic array (Electrocap Inc). The array is shown in Supplementary Fig. S9. EEG signals were amplified using a BioSemi ActiveTwo bioamplifier (https://www.biosemi.com/products.htm). External electrodes were placed on the outer canthi and under each eye to capture horizontal eye movements and blinks, respectively. All electrode offsets were kept under 20 millivolts. The data were sampled at 256 Hz (2048 Hz with a decimation factor of 1/8) and with a fixed first order analog antialiasing filter (−3 dB at 3.6 kHz).

Offline pre-processing and analyses were conducted using EEGLAB, ERPLAB and RStudio. The reference was set after data acquisition to the average of two electrodes placed on each mastoid. Data were filtered between 0.1 and 30 Hz. Data were segmented into epochs surrounding each target word, starting 100 ms before target word onset and ending 600 ms after.

Artifact-free epochs were obtained by removing components associated with blinks and horizontal eye movements through independent component analysis (ICA)^36,37^. Following ICA, epochs were inspected visually for eye movements and drifts to determine individualized thresholds. Automatic artifact rejection algorithms were applied to exclude epochs that did not meet these thresholds. Two Butterworth digital filters were applied to the data: a high-pass (low-cutoff) filter at 0.1 Hz and low-pass (high-cutoff) filter at 30 Hz. N400 waveforms were computed by averaging artifact-free epochs surrounding the statement-final target word as a function of statement type (pro-Democrat vs pro-Republican statements) and political issue dimension (economy, immigration, societal).

Participants with no fewer than 14 artifact-free epochs in the relevant conditions were retained for analyses. Five participants fell beneath the 14-trial threshold and were excluded from the computation of issue dimension-specific N400 effects. Of these, 3 were decided Republicans, 1 was a decided Democrat and 1 was undecided. On average, participants had 86 valid trials for Republican statements (range: 40–92) and 87 valid trials for Democratic statements (range: 39–92). For Economy issues, participants had 22 valid Republican (range: 18–23) and 22 Democratic trials (range: 21–23); for Immigration issues, 17 Republican (range: 14–18) and 17 Democratic trials (range: 16–18); for Societal issues, 26 for Republican (range: 22–27) and 27 Democratic statements (range: 23–30).

#### EEG analyses

To determine the onset of the effect of interest, the average peak latency of the N400 was calculated across all trials and all participants to determine the time window considered for statistical analysis. The N400 latency was established as the time at which the ERP difference between Republican and Democratic statements was maximal in the 250—600 ms period (to exclude the early visual potentials). The N400 peaked at 378 ms. The measurement window for the statistical analyses was centered around this peak value: 278–478 ms for all contrasts.

**The overall N400 effect** is the difference between the mean amplitude measurements post target word for all Republican statements minus all Democratic statements. These include statements on all issues: the Economy, Immigration, Societal Issues and general issue statements. The electrodes that displayed the greatest N400 effect were collapsed to compute these mean amplitude measurements.

N400 effects were calculated by subtracting the amplitudes for Democratic statements from those for Republican statements for each participant. Conceptualized this way, more positive values indicated greater agreement with Republican views while more negative values indicated greater agreement with Democratic views. Voters with a Republican leaning should show larger N400 responses to Democratic rather than Republican statements, resulting in positive N400 effects. The reverse was expected for voters with a Democratic leaning. In addition to the overall N400 effect, separate logistic regressions were run with the N400 effect corresponding to each separate issue dimension (Economy, Immigration, Societal).

#### ERP data analysis plan

Our ERP data analysis plan was threefold. It comprised (1) an omnibus mixed model ANOVA to test whether an N400 response occurred, (2) a series of logistic regressions to compare the predictive ability of our different implicit and explicit predictors and (3) a series of likelihood ratio tests to test whether the inclusion of ERPs to predictive models increased their predictive power.

We conducted an omnibus mixed model ANOVA with Vote Choice (two levels: Democrat, Republican) as a between-subjects factor and within-subject factors of Statement Type (Democrat, Republican), Issue Dimension (Economy, Immigration, Societal), and Electrode (26 levels). We carried out separate, follow-up ANOVAs for each group and Statement Type as part of an exploratory analysis. Analyses were implemented in R using the afex package which applies the Greenhouse-Geisser correction in case of violations of the assumption of sphericity. The post-hoc tests we used are pairwise t-tests run on every combination of the different factor variables. We applied the Benjamini-Yekutieli correction to the post-hoc t-tests’ significance levels. The ANOVA allowed us to test our hypothesis that the N400 effect aligns with political preferences and voting choice. We tested this by seeing whether the ANOVA showed a significant three-way interaction between Vote Choice (i.e. group), Statement Type and Electrode.

The logistic regressions and likelihood ratio tests allowed us to test our hypothesis that implicit measures of political preference are better predictors of undecided voters’ vote choices than explicit measures. We tested this hypothesis by seeing which variables were significantly predictive of voters’ voting choice.

We conducted likelihood ratio tests to examine whether adding the N400 effect to models with measures of explicit political preference improved their ability to predict vote choice. Likelihood ratio tests compare two models that are identical except for one predictor which is present in one model but not the other. If the test yields a significant chi-square value, it indicates that the additional model complexity resulting from adding the additional predictor is warranted.

The different voter groups were incorporated into the analyses by following Galli et al.’s 2021 analysis^10^, with Vote Choice as the between-subjects factor. The groups we compared were Democratic voters and Republican voters -regardless of whether they were decided or undecided—to examine whether the N400 effect aligned with differences in explicit political preference and voting choice.

We also compared decided Democrats and decided Republicans. If the N400 effect did reflect differences between decided voters, our plan was to replicate Galli et al.’s 2017^9^ analysis which tested if the N400 effect of undecided voters did not differ from that of the decided voters who voted for the same party as them. For example, to test if the N400 effect of undecided voters who voted Democrat did not differ from the N400 effect of decided Democrats.

## Results

### Explicit preference index (EPI)

**Figure 1:**
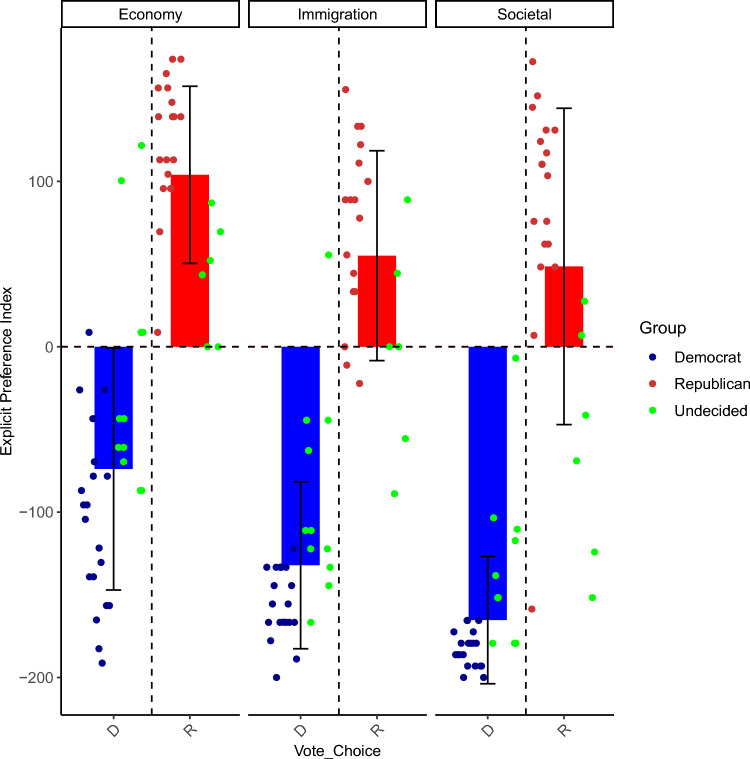
EPI scores for Democratic (D) and Republican (R) voters across 3 issue dimensions. The same individuals are plotted for each issue.

Figure 1 shows the mean EPI for the different issue dimensions of economy, immigration and societal statements for participants who voted Democrat or Republican. More positive values indicate greater agreement with Republican views while more negative values indicate greater agreement with Democratic views. Figure 1 shows that participants who voted Republican were more strongly aligned with the Republican party stance on Economic issues (m = 104, range: − 61, 163) than participants who voted Democrat with the Democratic party stance (m = − 73.9, range: − 183, 52). Participants who voted Democrat were more aligned with the Democratic party stance on Immigration (m = − 132.1) and Societal statements (m = − 165.3) than participants who voted Republican with the Republican party stance on those issues (m = 55.1 and m = 48.6, respectively). Participants who voted Democrat had less variance in their EPIs on immigration and societal issues compared to participants who voted Republican whose explicit preferences on those issues varied considerably. This was less the case with economic issues, for which participants who voted Republican had less variance than participants who voted Democrat. Overall, participants who voted Republican were less aligned with the Republican party stance (m = 67.12, range = − 60.87, 163.04) than participants who voted Democrat (m = − 127.37, range = − 182.61, 52.17).

An ANOVA was conducted with EPI as the dependent variable, Vote Choice (two levels: Democrat, Republican) as the between-subjects factor, and Issue Dimension (three levels: Economy, Immigration, Societal) as the within-subjects factor. The counts in each cell were as follows: for Vote Choice, 31 participants voted Democrat and 24 Republican; for Issue Dimension, there were three different EPI values per participant, one for Economy statements, one for Immigration and one for Societal issues. There was a significant main effect of Vote Choice (F_1,53_ = 163.62, p < 0.001, ges = 0.699) and Issue Dimension (F_1.95, 103.27_ = 51.29, p < 0.001, ges = 0.193). Our post-hoc analyses in Table 1 show that the main difference in EPI between issues was between the economy on the one hand and immigration and societal issues on the other, with economy issues receiving less negative scores than the other two issue dimensions. The lack of an interaction with vote choice (F_1.95, 103.27_ = 3.1, p = 0.51, ges = 0.014) indicates that the vote choice groups differed equivalently on all Issue Dimensions.

**Table 1:** Means table of EPI scores by ANOVA contrasts.

	Economy	Immigration	Societal
Average EPI	3.72	− 50.43	− 71.98
Democrats	− 73.9 (13.15)	− 132.1 (9.06)	− 165.3 (6.91)
Republicans	104 (10.94)	55.1 (12.96)	48.6 (19.53)

### Waveform morphology

**Figure 2:**
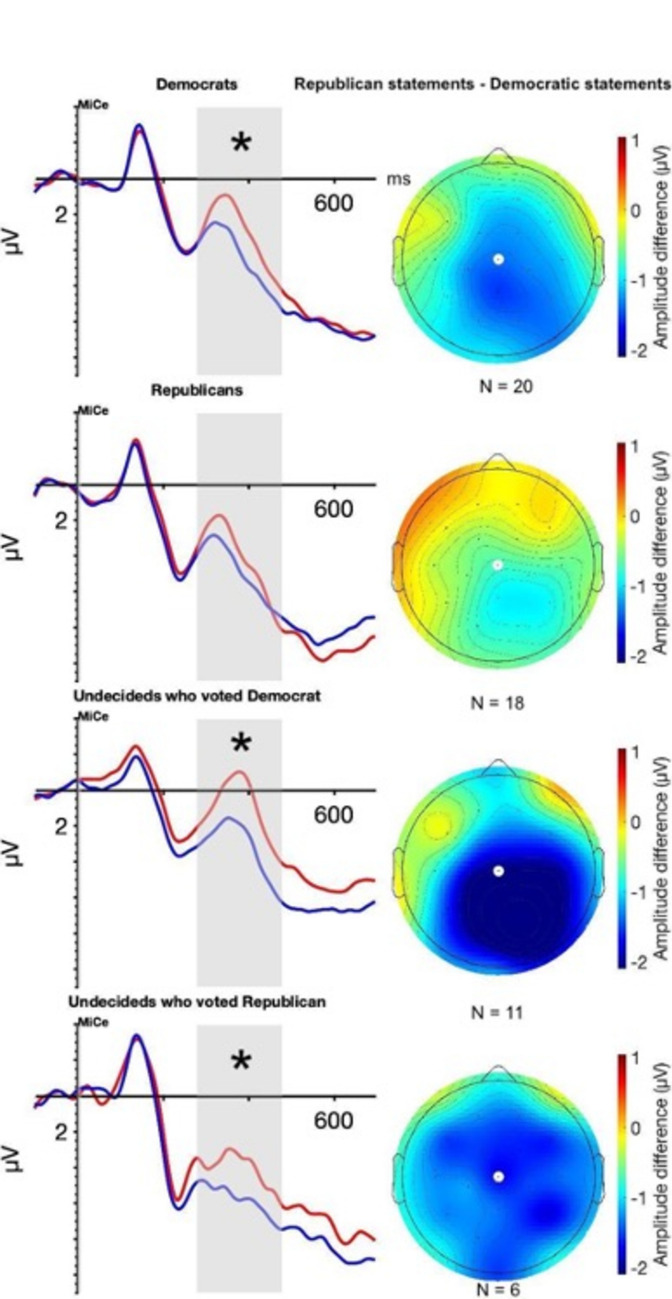
N400 in voters who voted Democrat or Republican for governor. The waveforms on the left show the grand averaged waveforms for statements with pro-Democratic (in blue) and pro-Republican (in red) target words. Significant Statement Type differences between Republican statements and Democratic statements are indicated by an asterisk. All waveforms are from the vertex electrode MiCe which is indicated by a white dot on the scalp maps. Grand averaged waveforms for all electrodes can be found in Supplementary Figs. S5, S6, S7, S8. The gray overlay shows the time window used for statistical analyses (278–478 ms). The scalp maps on the right show the scalp distribution of the ERP difference (Republican - Democratic statements) as isovoltage in microvolts.

Figure 2 shows the grand-average ERPs from the vertex electrode (MiCe) time-locked to the statement-final word. Sensory components in the visual evoked potential (VEP) were followed by a negative-going deflection around 400 ms following stimulus onset with larger amplitude for Republican statements than Democratic statements.

### N400 mean amplitude

Our first step was to see if we could replicate Galli et al.’s findings by seeing if Democratic and Republican voters presented opposite N400 patterns. Following the literature, the N400 for statements that violate one’s political beliefs should be greater than for statements that support them. For Democratic voters, the N400 was therefore expected to be more negative for Republican statements than for Democratic statements. Inversely, for Republican voters, the N400 was expected to be more negative for Democratic statements than for Republican statements. Figure 2 shows the expected pattern for Democratic voters but not Republican voters who do not show the inverted N400 pattern. Undecided voters, whether they ultimately voted Republican or Democrat, show an N400 effect similar to the Democratic voters.

Democratic voters showed larger N400 amplitude to Republican statements than Democratic statements. Decided Republican voters showed no difference in the N400 response to Republican and Democratic statements, as the interaction between Statement Type and Electrode (F_25, 575,_ = 1.51, p = 0.054, η_p_^2^ = 0.001) is not significant when only looking at Republican voters. On the whole, Republican statements led to larger N400s.

**Figure 3:**
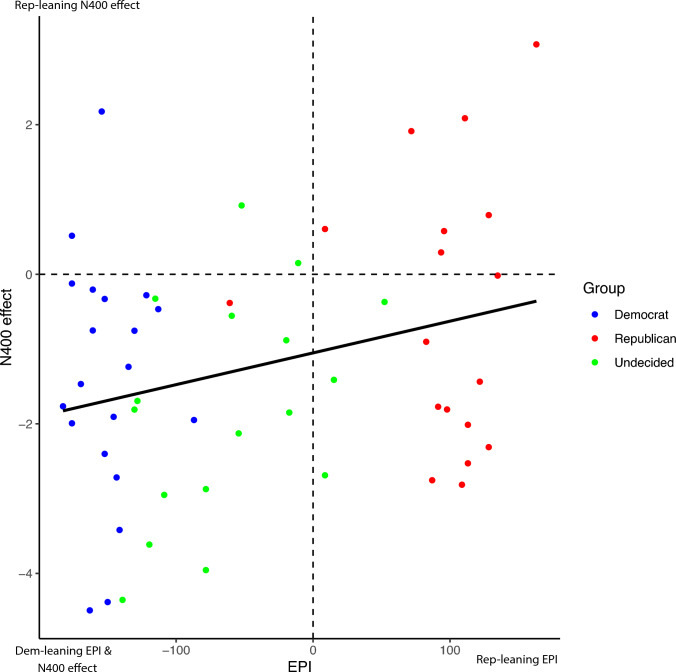
Individual participants are shown with their respective N400 effect values (in µV) and EPI scores. The black line shows the regression line when regressing the N400 effect on EPI score. The effect of the EPI score on the N400 effect is positive and significant (p = 0.0382, t = 2.125). The breakdown of these data by issue dimension can be found in Supplementary Figs. S10, S11, S12.

Figure 3 shows the positive and significant relationship between the N400 effect and EPI scores. If participants’ explicit political preferences are aligned with their implicit political preferences measured by the N400 effect, dots should either be in the bottom left quadrant for Democrats or in the top right quadrant for Republicans. Although a majority of participants fall in these two quadrants, 12 participants with Republican-leaning EPI scores presented Democratic-leaning N400 effects. These are shown in the bottom-right quadrant. Of these 12 participants, 9 were decided Republicans and 3 were undecided. Similarly, 4 participants with Democratic-leaning EPI scores presented Republican-leaning N400 effects, as shown in the top left quadrant. Of these, 2 were decided Democrats and 2 were undecided.

As previously explained, we conducted an omnibus mixed model ANOVA with Vote Choice as a between-subjects factor and within-subject factors of Statement Type, Issue Dimension and Electrode.

The main effect of Vote Choice did not reach significance (F_1, 48_ = 1.57, p = 0.217, η_p_^2^ = 0.013), indicating that the N400 amplitudes to all target words did not differ overall between Republican and Democratic voters. This is expected given that voters read both statement endings. There were significant main effects of Statement Type (F_1, 48_ = 23.93, p < 0.001, η_p_^2^ = 0.017), Issue Dimension (F_2.72, 130.65_ = 4.03, p = 0.011, η_p_^2^ = 0.007), and Electrode (F_2.91, 139.86_ = 27.11, p < 0.001, η_p_^2^ = 0.130). These effects revealed that N400 amplitude was more negative overall for Republican (1.60 µV) than Democratic (2.44 µV) statements and for Immigration statements (1.64 µV) than Economy and Societal statements (respectively 2.18 and 2.44 µV).

These main effects were qualified by three interactions.

The Statement Type by Electrode interaction was significant (F_5.21, 250.07_ = 9.99, p < 0.001, η_p_^2^ = 0.003). Post-hoc analysis revealed that the N400 effect, with larger amplitude for Republican than Democratic statements, was largest over central and parietal electrodes, as is typical for an N400 effect to words.

Issue Dimension by Electrode was significant (F_75, 3600_ = 1.55, p = 0.002, η_p_^2^ = 0.001), but appears to be due to chance as it only concerns one Electrode x Issue Dimension contrast out of 156 contrasts (Immigration statements generated more negative amplitudes than Societal statements at RDCe).

A Statement Type by Issue Dimension interaction (F_2.76, 140.98_ = 1.84, p < 0.001, η_p_^2^ = 0.002) indicated that Democratic and Republican statements differed in N400 amplitude only for Immigration and Societal issues, but not Economic statements, as shown in Table 2. N400 amplitudes for Immigration and Societal statements were more negative-going when they were Republican than Democratic. This happened regardless of Vote Choice given that there was no 3-way interaction with Vote Choice (for a breakdown of N400 responses by Issue Dimension, see Supplementary Figs. S1, S2, S3).

**Table 2:** Mean amplitude: Statement type by issue dimension.

Issue dimension	Economy (µV)	Immigration (µV)	Societal (µV)
Democratic statements	2.22	2.2	3.02
Republican statements	2.16	1.08	1.86

There was no significant interaction between Vote Choice and Statement Type (F_1, 48_ = 0.96, p = 0.331, η_p_^2^ < 0.001) and between Vote Choice and Issue Dimension (F_2.72, 130.65_ = 0.93, p = 0.422, η_p_^2^ = 0.002). This indicates that there was no significant effect of the voting group participants belonged to on the N400 effect. We obtained the same results when limiting our analysis to decided voters. Even in this case, there was no significant interaction between Vote Choice and Statement Type (F_5.21, 250.07_ = 1.61, p = 0.155, η_p_^2^ < 0.001). Because the N400 effect did not reflect differences between decided voters, we did not test whether undecided voters’ N400 effects were similar to those of decided Democrats or decided Republicans, as in Galli et al.’s 2017 analysis^9^.

We further explored the interaction between Statement Type and Electrode by means of a distributional analysis, with Statement Type, Anteriority (4 levels: prefrontal, frontal, parietal, occipital), Laterality (2 levels: lateral, medial) and Hemisphere (2 levels: left, right) as within-subject factors. Electrodes used in the distributional analysis are shown in gray on Supplementary Fig. S9. This analysis found a maximum N400 effect (Republican statements versus Democratic statements) over right hemisphere medio-central electrodes (statement Type by Anteriority, F_1.36, 73.56_ = 5.59; statement Type by Laterality, F_1, 54_ = 17.21; statement Type by Hemisphere, F_1,54_ = 4.04). The timing and scalp distribution of the ERP waveforms are consistent with a typical N400 effect, which is known to be greatest at central-parietal sites and to peak around 400 ms after stimulus onset^13^.

## Valence

As a further check, we examined whether differences in valence between sentence-final target words across Statement Type conditions may have impacted N400 amplitudes. Some of our sentence-final target words vary in valence. For example, the word “patriot” and “thug” have different emotional valences. Unless controlled for across the two conditions, differences in valence could have potentially affected N400 differences between groups and represented a potential confound that could have led to no differences in the N400 effect across groups.

To determine whether differences in valence affected N400 differences between groups, we checked whether a difference in valence existed between stimuli across the two conditions. In other words, whether pro-Democratic and pro-Republican statements presented a difference in valence. To do this, we consulted a database with valence scores for nearly 14,000 English words^38^. The database provided valence scores for 163 out of 184 stimuli. 10 stimuli were proper names like “Biden” and “Abbott” and do not have valence ratings in published norms. 11 stimuli were not present in the database and are reported in the supplementary information. The database gives the dictionary, singular form for nouns and the infinitive form for verbs. It does not contain adverbs so we used the valence of the corresponding adjective instead (e.g. using the valence of “appropriate” for the stimulus “appropriately”). We ran an ANOVA with Statement Type as between-subjects factor and Valence score as dependent variable. There was no significant main effect of Statement Type on Valence and hence no overall difference in Valence between pro-Democratic and pro-Republican statements (F1, 160 = 3.09, p = 0.081, ηp2 < 0.019). As there was no difference in Valence across conditions, Valence cannot explain the absence of N400 differences between groups. We include the valence scores of our stimuli in the Supplementary Information.

### Logistic regressions

We turn to the question of whether the N400 effect is a significant predictor of voting behavior and how it compares to other variables. We inserted each of the variables described in the Predictors section as the single predictor of a logistical regression predicting Vote Choice. We set the reference category of Vote Choice to “Republican”. The coefficients in Table 3 show the changes in log odds of voting Democrat. For the N400 effect variables, we used the absolute average voltage of the subset of electrodes where the N400 effect was greatest. These electrodes were determined on the basis of a visual inspection of the scalp maps and included MIPa, MiCe, LMCe, RMCe, LMOc, RMOc, LDPa and RDPa.

**Table 3:** Logistic models.

	(1)	(2)	(3)	(4)	(5)	(6)	(7)	(8)
Child-rearing authoritarianism	− 0.576 **							
	(0.197)							
VSAS		−0.347 ***						
		(0.101)						
Economy perception			1.182 *					
			(0.497)					
Biden job approval				3.330 **				
				(1.083)				
Top issue: societal issues					2.680 ***			
					(0.755)			
Explicit preference index						− 0.041 **		
						(0.012)		
N400 effect							− 0.313	
							(0.176)	
N400 effect—economy								0.05
								(0.09)
N	55	55	55	55	52	55	55	50
AIC	68.209	61.331	72.723	60.806	58.399	22.446	75.894	73.967

Table 3 shows the coefficients and standard errors (in parentheses) of the single-predictor logistic models, with their different levels of significance.

***p < 0.001; **p < 0.01; *p < 0.05.

The overall N400 effect (p = 0.07, z = − 1.782), which included all statements, did not predict voting behavior at the 0.05 level of significance, nor did the N400 effects for Immigration (p > 0.1, z = 0.94), Societal (p > 0.1, z = 0.56) or Economy (p = 0.067, z = 1.62) Statements. This is consistent with the fact that we did not find the N400 patterns we were expecting to replicate from Galli et al.’s studies. Because the direction of the N400 effect did not co-vary with voting differences, the N400 effect was not a significant standalone predictor of Vote Choice.

The Explicit Preference Index (p < 0.01, z = − 3.290), had a high level of significance in predicting Vote Choice, as did its components, the EPIs for Economy (p < 0.001, z = − 3.935), Immigration (p < 0.001, z = − 3.732) and Societal (p < 0.001, z = − 3.605) issues. Voting Intention was also a significant predictor of actual Vote Choice (p < 0.001, z = -3.767).

Our two variables measuring authoritarianism, child-rearing values (p < 0.01, z = − 2.915) and VSAS (p < 0.001, z = − 3.421), were both predictive of voting behavior. Economy perception (p < 0.05, z = 2.379) also predicted voting behavior, with voters who thought the state of the economy was ‘Good’ or ‘Only Fair’ more likely to vote Democrat than Republican. Positive approval ratings of the president—who at the time of the election was Joe Biden, a Democrat—significantly increased the likelihood of voting Democrat (p < 0.01, z = 3.073). Gender was a significant predictor, with women more likely than men to vote Democrat (p < 0.05, z = 2.306), although this gender difference is likely to be due to sampling given the relatively small sample size per group and the higher proportion of women among decided Democrats compared to the other groups.

The variable ‘most important problem facing the country’ (p < 0.001, z = 3.550) was predictive of voting behavior: voters for whom the top issue facing the country was societal issues (abortion, race relations, gun rights) were more likely to vote Democrat than voters for whom the top issue was the economy.

Political knowledge (p > 0.05, z = − 0.134) and race and ethnicity (p > 0.05, z = {− 0.989: 1.399}) did not predict voting behavior. The latter could be due to the high number of levels (9) in the race/ethnicity factor, with unequal numbers across the different levels.

### Likelihood ratio tests

Following Galli et al.’s approach, we examined whether adding the N400 effect to the EPI improved model fit. We ran likelihood ratio tests comparing a model with only the EPI to a model with both the EPI and the different types of N400 effect (overall, Economy, Immigration, Societal). The test showed that adding the N400 effect for Economy issues to the EPI significantly reduces deviance from 17.39 to 12.78, as shown in Table 4. The fact that the test is significant indicates that the additional model complexity resulting from adding the N400 effect for Economy statements as a predictor is warranted. Adding the overall N400 effect or the N400 effect for Immigration statements or the N400 effect for Societal statements to the EPI did not significantly reduce deviance compared to the model with only the EPI.

**Table 4:** Analyses of deviance.

	Resid. Df	Resid. Dev	Df	Deviance	Pr(> Chi)
EPI model + N400 economy					
EPI model	48	17.392			
EPI + N400 economy	47	12.777	1	4.615	0.03169*
EPI model + overall N400					
EPI model	48	17.392			
EPI + overall N400	47	17.355	1	0.037145	0.8472
EPI model + N400 societal					
EPI model	48	17.392			
EPI + N400 societal	47	17.294	1	0.098027	0.7542
EPI model + N400 immigration					
EPI model	48	17.392			
EPI + N400 immigr	47	15.066	1	2.3257	0.1273

## Discussion

The goal of this study was twofold: to see if the N400 effect reflected differences in political alignment and if this and another implicit measure of political preference, namely authoritarianism as measured by the child-rearing values scale, could predict vote choice above and beyond explicit voter preferences. The research hypotheses were based on previous work with European voters^9,10^ and were tested in young adult voters in the US. Under the assumption that the N400 effect would reflect vote choice and explicit political preferences in decided voters, we predicted that implicit measures of political preference would better predict undecided voters’ vote choice than explicit measures of political preference.

Our data show that the explicit measures of political preference performed well in predicting vote choice. The EPI, the president’s job approval, the VSAS and “the most important issue” predictor were all significantly predictive of vote choice. In addition, our second implicit measure of political preference, authoritarianism as measured by the child-rearing scale, was predictive of vote choice, with higher scores indicating a greater probability of voting Republican. As mentioned in the introduction, authoritarianism has been shown to predict electoral swings in different countries. Stenner and Haidt^24^ showed that individuals with high authoritarianism scores were considerably more likely to vote for far-right parties in conditions of high normative threat. Our implicit measure of authoritarianism (the child-rearing scale) did not predict vote choice above and beyond our explicit measure of authoritarianism (the VSAS) or above and beyond the other explicit measures of political preference (e.g. EPI, top issue facing the country) as these were also highly significant predictors of vote choice and had similarly substantial effects. However, it offers a valuable insight. Although our sample was not polarised on certain issue dimensions (Democrats on the Economy, Republicans on Immigration and Societal issues), Republican and Democratic voters were split along the dividing line of the social attitudes measured by authoritarianism, which are conformity to in-group norms and deference to political leaders. Future research could ascertain whether this factor was the key influence in shaping the vote choice of undecided voters who voted to the right, of which we had too small a sample (N = 6) to draw inferences from.

However, our results for the N400 effect differed from what was previously observed in Italian and British voters in Galli et al.’s findings. Galli et al. found that the N400 effect aligned with vote choice in decided voters, with Remain voters presenting larger negativities for Leave compared to Remain statements and Leave voters displaying the opposite, with larger negativities for Remain compared to leave statements. In our study, we expected Democratic voters to display larger N400 amplitude for pro-Republican compared to pro-Democratic statements, and for Republican voters to display the reverse pattern, with larger negativities for pro-Democratic compared to pro-Republican statements. We did find this with Democratic voters but not with Republican voters. There was no significant group difference in N400 patterns, with pro-Republican statements eliciting larger N400 amplitude than pro-Democratic statements in both Republican and Democratic voters. Follow-up ANOVAs suggested that decided Republicans showed no statistical difference between statement types. The undecided voters as a group displayed a similar pattern to (not significantly different from) decided Democratic voters, with larger responses to pro-Republican than pro-Democratic endings. However, not all undecided voters chose to vote Democratic (11 voted Democrat and 6 Republican).

Critically, these findings do not compromise the use of the N400 to detect preferences in undecided voters because preferences and actual behavior are distinct phenomena. The N400 effect failed to predict the behavior, which is voting. However, the evidence does not suggest that it failed to predict preferences, specifically implicit preferences. Rather, it would seem that it detected implicit preferences and those preferences happened to be different from explicit preferences among decided Republicans.

Other research has also shown dissociations between behavioural measures and brain activity. McLaughlin et al.^39^ for example suggested that behavioral assessments of second language learning underestimated the amount learned by participants. They argued that “ERPs might more accurately reflect implicit learning and continuous change in knowledge than do explicit, categorical judgments”. This did not entail that their implicit (neural) or explicit (behavioral) measures were invalid. Cerda et al.^40^ also observed a dissociation between behavioral measures and ERPs. Although the object of research in these studies was language learning and bilingual cognition, the same is likely to apply to political judgments and attitudes. Arguably, ERPs more accurately reflected implicit preferences, even if explicit preferences ultimately weighed more than implicit preferences in shaping voting choices. Furthermore, if behavioural and neural measures covaried perfectly and accounted for exactly the same variance, there would be no purpose in recording implicit measures like ERPs.

It is important to note that even in the Galli et al. studies, the N400 effect was not always predictive of voter behavior. Galli et al.^10^ found that the N400 effect for economic issues was predictive of Italian voters’ choices, while the N400 effects for cultural and anti-establishment issues were not. Our logistic models show that neither the overall N400 effect nor the issue-specific N400 effects were predictive of vote choice when inserted as single predictors in a logistic regression. However, inclusion of the N400 effect for Economy in more complex models did improve the predictive ability of measures of explicit preference, like the EPI. This suggests that it might be explaining a portion of variance corresponding to implicit preferences which the EPI does not account for.

This finding is especially notable given that participants were less polarised on the Economy than on Immigration or Societal issues (see Supplementary Figs. S10, S11, S12), yet the N400 effects for Immigration and Societal issues were not predictive and did not improve the predictive ability of measures of explicit preference. One speculative explanation for this could be that the EPI scores for Immigration and Societal issues already accounted well for the variance relative to those issue dimensions, which meant that the N400 effect could not add much to those EPI variables’ predictive ability. For the Economy, on the other hand, the N400 effect may have added more to the EPI’s predictive ability because participants’ explicit preferences were less polarised on the Economy and therefore the EPI may have accounted for less variance on the Economy than it did on Immigration and Societal issues.

The fact that Republican voters did not present larger N400 amplitude for pro-Democratic compared to pro-Republican statements could have different explanations. This finding may be surprising given that the US is characterized by a more polarized political context^41^ than the European countries the two Galli et al. studies were set in and where N400 responses were inverted for participants from opposing political sides. Although notably, data from the EPI scores suggest that our sample of decided Republicans were less polarized than our sample of decided Democrats. The lack of difference in N400 responses to pro-Democratic and pro-Republican statements among decided Republicans is consistent with the fact that this sample of Republican voters was less polarized as a group. In other words, the N400 effect, as an implicit measure of political preferences, reflects that this sample on average was less consistently polarized to the statements than the decided Democrats in our sample.

The context in which the 2022 midterm elections took place is another possible explanation. The elections took place a few months after the Supreme Court of the United States overturned Roe versus Wade, which had enshrined women’s right to abortion since 1973. Although the congressional Republican party had been actively pursuing this outcome for several years, a sizable minority of Republican voters (approximately 30% at that time^42^) opposed it. The N400 effects we observed among Republican participants might have been partially due to the fact that some of them had conflicting or at the least mixed explicit preferences, although they voted for the Republican party.

Among our participants, most Republican voters’ brain responses suggested agreement with Democratic statements, according to the N400 effect. Yet, membership of a political group and explicit preferences seem to have been the key factors behind vote choice rather than the implicit preferences measured by the N400 effect. Recent accounts^43^ have found that group membership and conformity with one’s in-group have been major determinants of electoral behavior in recent years. The fact that the N400 effect was not predictive of vote choice could suggest that conformity with in-group values was more important than individual responses to policy statements (as measured by the N400 effect) when determining one’s vote.

The EPI scores reported in Fig. 1 support this interpretation to a certain extent. Republican voters had greater variance on Immigration (sd = 63.49) and Societal issues (sd = 95.66) than Democrats (sd = 50.47 for Immigration and sd = 38.49 for Societal issues), whose EPI scores showed considerable homogeneity on those issues. Some Republican voters’ EPI scores on Immigration and Societal issues were in fact aligned with the Democratic party stance, as indicated by the data points under the Republican bars scoring negatively (which indicates alignment with the Democratic party stance). This was not the case with Economy issues where Republican voters (sd = 53.57) showed greater homogeneity in their EPI scores than Democratic voters, whose EPI scores were more spread out (sd = 73.23) and, for some participants, were aligned with the Republican party stance on Economic issues. The fact that group membership -often referred to as partisan affiliation- had a greater influence in shaping vote choice than the individual implicit preferences measured by the N400 effect could be a reflection of the importance of group membership for American voters.

It is also possible for the context in which data acquisition occurs to influence participants’ neural responses. Previous studies have shown that participant expectations, which develop during an experiment as a result of the experimental manipulation, can influence N400 responses. Several studies have shown that the N400 is reflective of contextual expectations^12,13^. The ERP patterns observed among Republican voters might have been influenced by their expectations given the experimental context (e.g. if they assumed that pro-Republican statements would be less common in a university environment) rather than solely driven by their own personal beliefs. However, this is unlikely given that the sentences presented were equally likely to end with a word that was pro-Republican or pro-Democrat. Participants would have quickly realized that the statements were not biased toward a more liberal perspective.

Moreover, there is evidence for participant acquiescence bias in different research contexts, although not using ERPs specifically^44,45^. Participants have been known in certain cases to bias their own answers to try to align with what they perceived the experimenter to want. If participants in our study perceived the university (i.e. the context in which the research occurred) as being “on their side”, belief-congruent statements could be matching participants’ predictions, whereas if they believe the university is against their views, belief-incongruent statements may be matching their predictions instead. However, we do not know if such acquiescence bias took place and can only speculate as to the direction in which it would have occurred. Critically, the N400 response is pre-conscious^46^. Perceived alignment or divergence with the university would be at least in part a conscious reasoning which cannot deliberately alter the N400 response.

Stimulus valence can also modulate the N400, with larger N400 amplitude for words that are negatively valenced than for positively valenced words^47–49^. In our data, pro-Democratic stimuli never elicited larger N400 amplitude in any of the groups (Democrat, Republican or undecided voters). One might argue that there could have been an overall difference in valence between statement types, leading the pro-Republican stimuli to be perceived as more negatively overall by all participants. However, we can rule this out as a possible explanation given that there was no overall difference in valence between pro-Democratic and pro-Republican statements.

In conclusion, we found that the N400 effect correlated with expressed policy preferences among Democratic voters but did not significantly co-vary with or independently predict voting differences between Democratic and Republican voters. The N400 effect showed a marked preference for pro-Democratic statements in Democratic voters and suggested ambivalence between pro-Democratic and pro-Republican statements in decided Republican voters. This finding was, to a certain extent, corroborated by the EPI, which showed Republican voters being less aligned with their party than Democratic voters were with theirs. Although the N400 effect did not behave in the way we predicted, it does appear consistent with the subtle differences in political preferences expressed explicitly through our EPI score. Contrary to our predictions, the N400 effect did not correlate with vote choice. Implicit measures like the child rearing authoritarianism scale currently show a clearer ability to predict voting behavior compared to the N400 effect. Given previous demonstrations^9,10^ in which the N400 effect was predictive, it is currently unclear why the N400 neural response might enable significant predictions in some contexts but not others. We originally anticipated that neural signals like the N400 effect might be even more predictive in highly polarized contexts like the US. Perhaps partisan identity-based voting means that this particular measure, which is dependent on semantic processing by each individual, is actually less able to predict voting decisions than group-wide preferences.

### Supplementary Information

Supplementary Information.

## Data Availability

The anonymised datasets generated during and analyzed during the current study are available from the corresponding author on reasonable request.
