# Supplementary material for: The N400 effect captures nuances in implicit political preferences
Source: Sci Rep. 2024 Jul 20;14:16730. doi: 10.1038/s41598-024-67763-7 (PMC11271581; doi:10.1038/s41598-024-67763-7)
Supplement: Supplementary file 1 — Supplementary Information. [file 41598_2024_67763_MOESM1_ESM.docx]

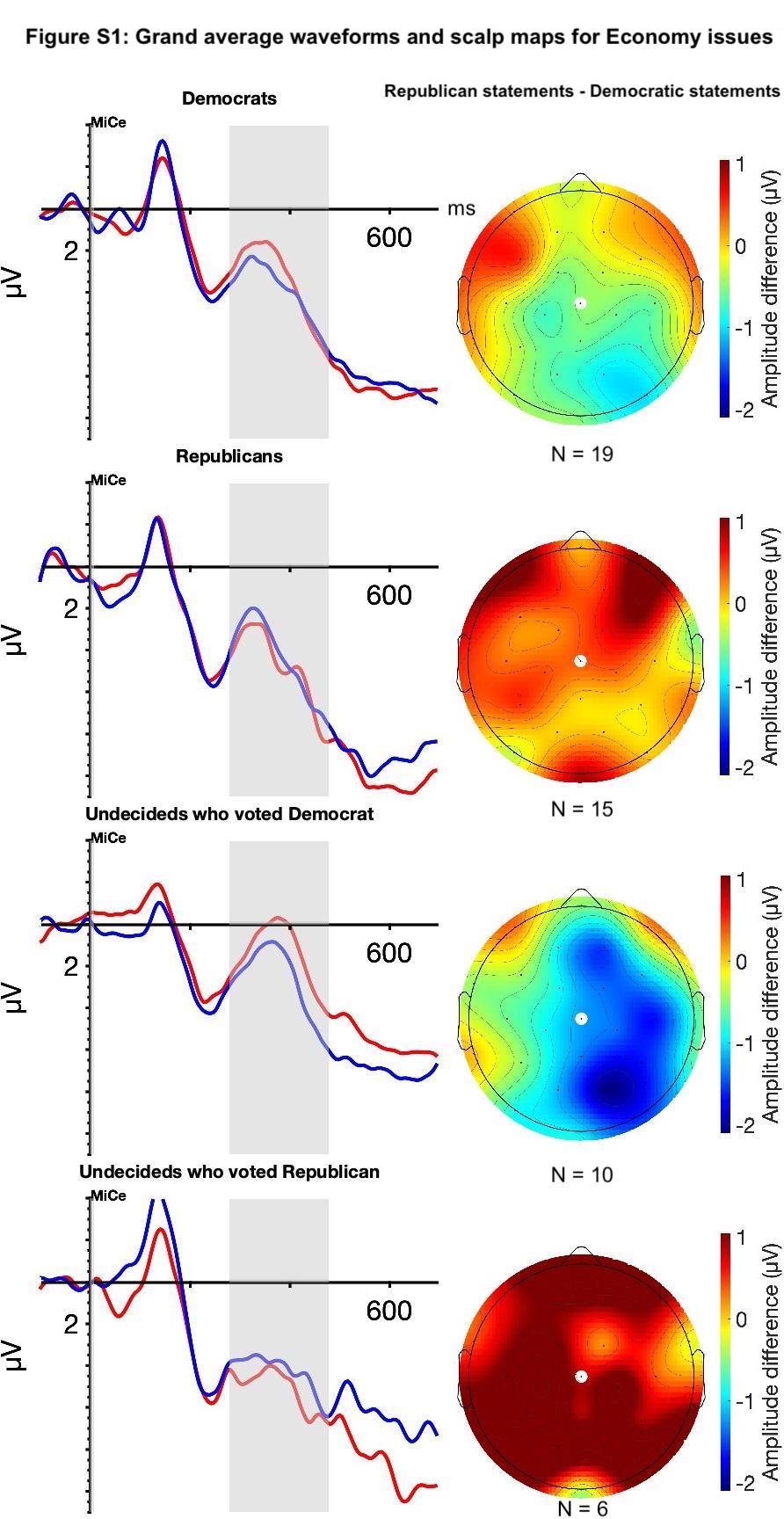


**Figure S1**. N400 response to statements on Economic issues. The waveforms on the left show the grand averaged waveforms for statements with Democratic (in blue) and Republican (in red) target words. All waveforms are from the vertex electrode MiCe which is indicated by a white dot on the scalp maps. The gray overlay shows the time window used for statistical analyses (278ms – 478 ms). The scalp maps on the right show the scalp distribution of the ERP difference (Republican - Democratic statements) as isovoltage in microvolts.


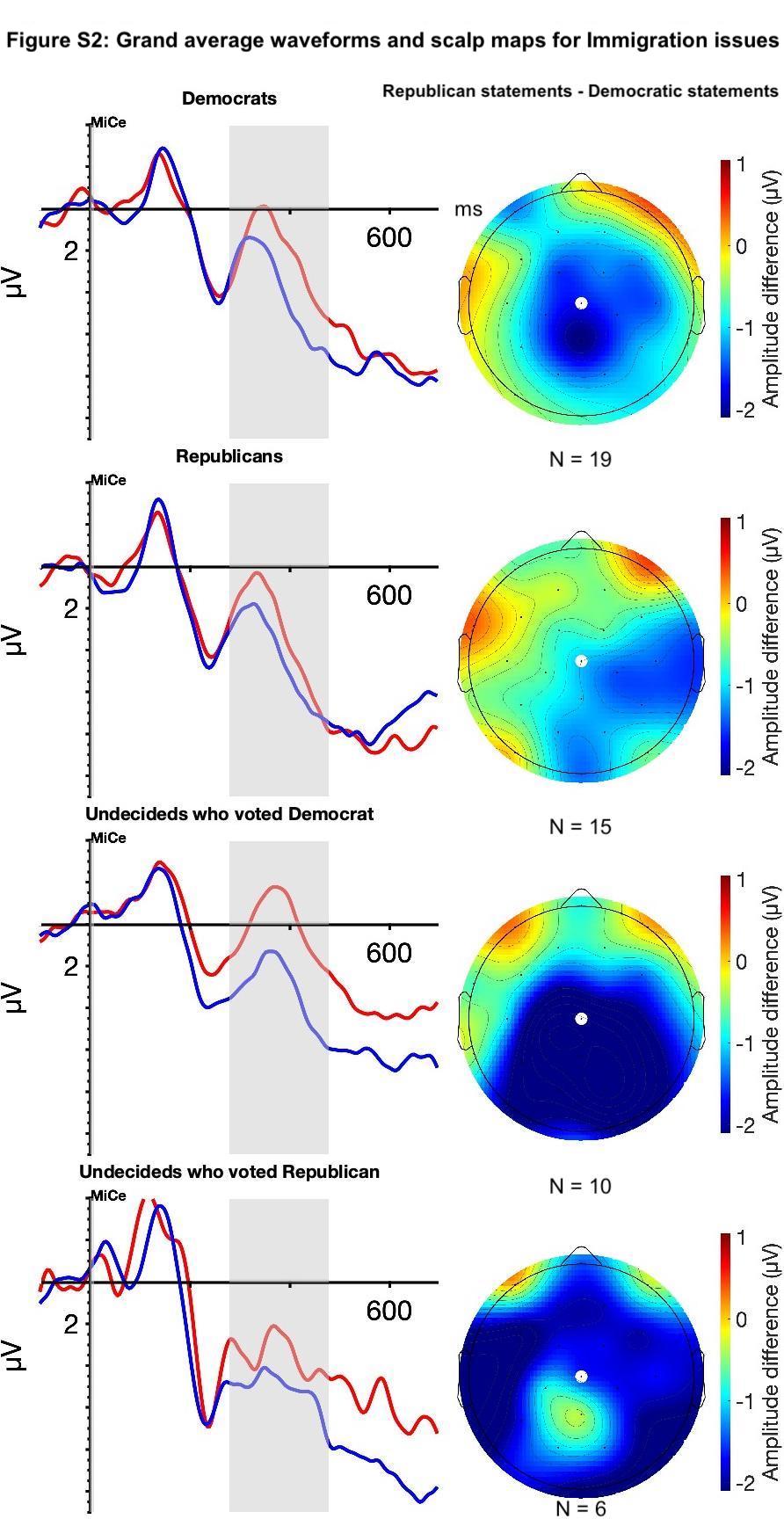


**Figure S2**. N400 response to statements on Immigration issues. The waveforms on the left show the grand averaged waveforms for statements with Democratic (in blue) and Republican (in red) target words. All waveforms are from the vertex electrode MiCe which is indicated by a white dot on the scalp maps. The gray overlay shows the time window used for statistical analyses (278ms – 478 ms). The scalp maps on the right show the scalp distribution of the ERP difference (Republican - Democratic statements) as isovoltage in microvolts.


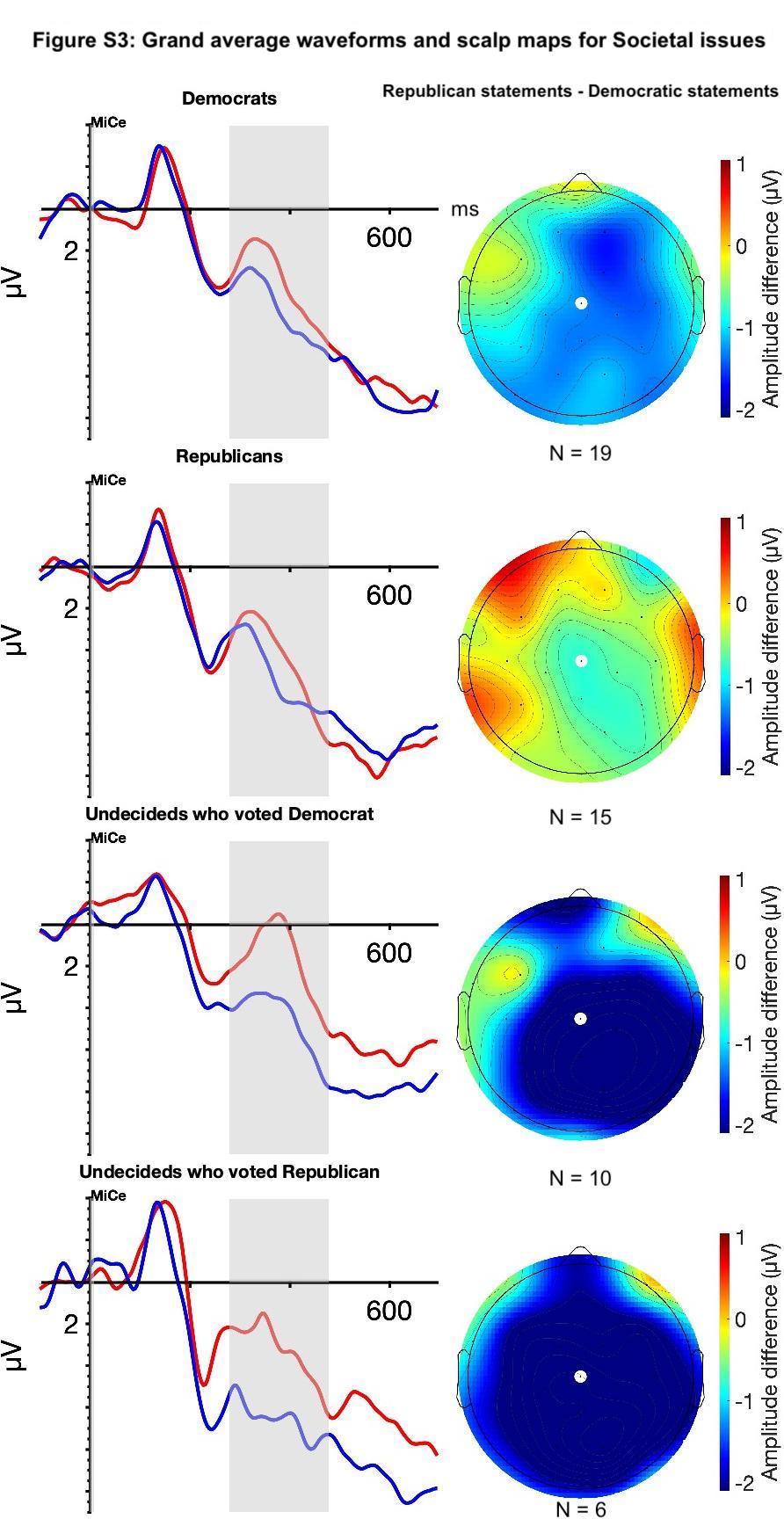


**Figure S3**. N400 response to statements on Societal issues. The waveforms on the left show the grand averaged waveforms for statements with Democratic (in blue) and Republican (in red) target words. All waveforms are from the vertex electrode MiCe which is indicated by a white dot on the scalp maps. The gray overlay shows the time window used for statistical analyses (278ms – 478 ms). The scalp maps on the right show the scalp distribution of the ERP difference (Republican - Democratic statements) as isovoltage in microvolts.


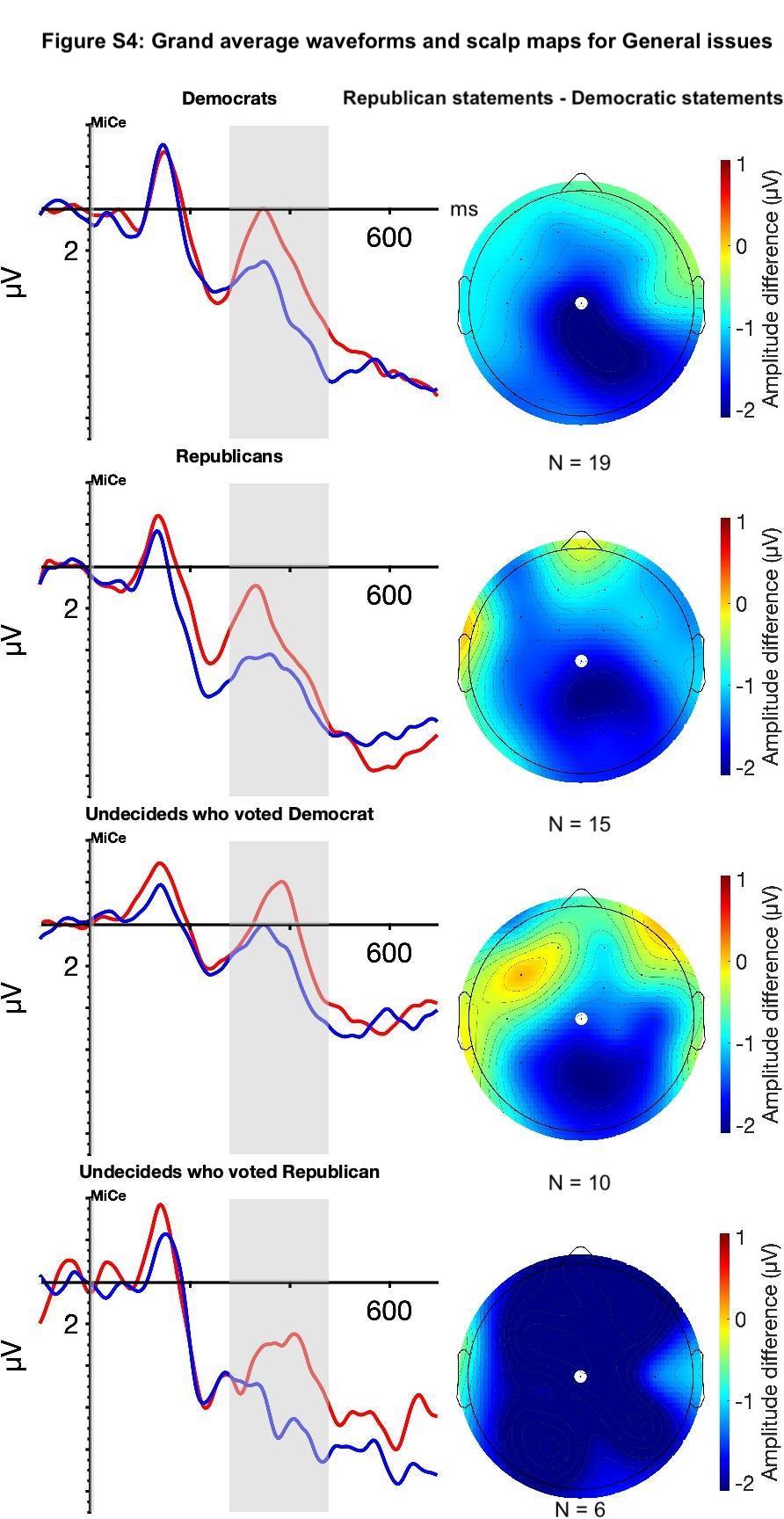


**Figure S4**. N400 response to statements on General issues. The waveforms on the left show the grand averaged waveforms for statements with Democratic (in blue) and Republican (in red) target words. All waveforms are from the vertex electrode MiCe which is indicated by a white dot on the scalp maps. The gray overlay shows the time window used for statistical analyses (278ms – 478 ms). The scalp maps on the right show the scalp distribution of the ERP difference (Republican - Democratic statements) as isovoltage in microvolts.

**
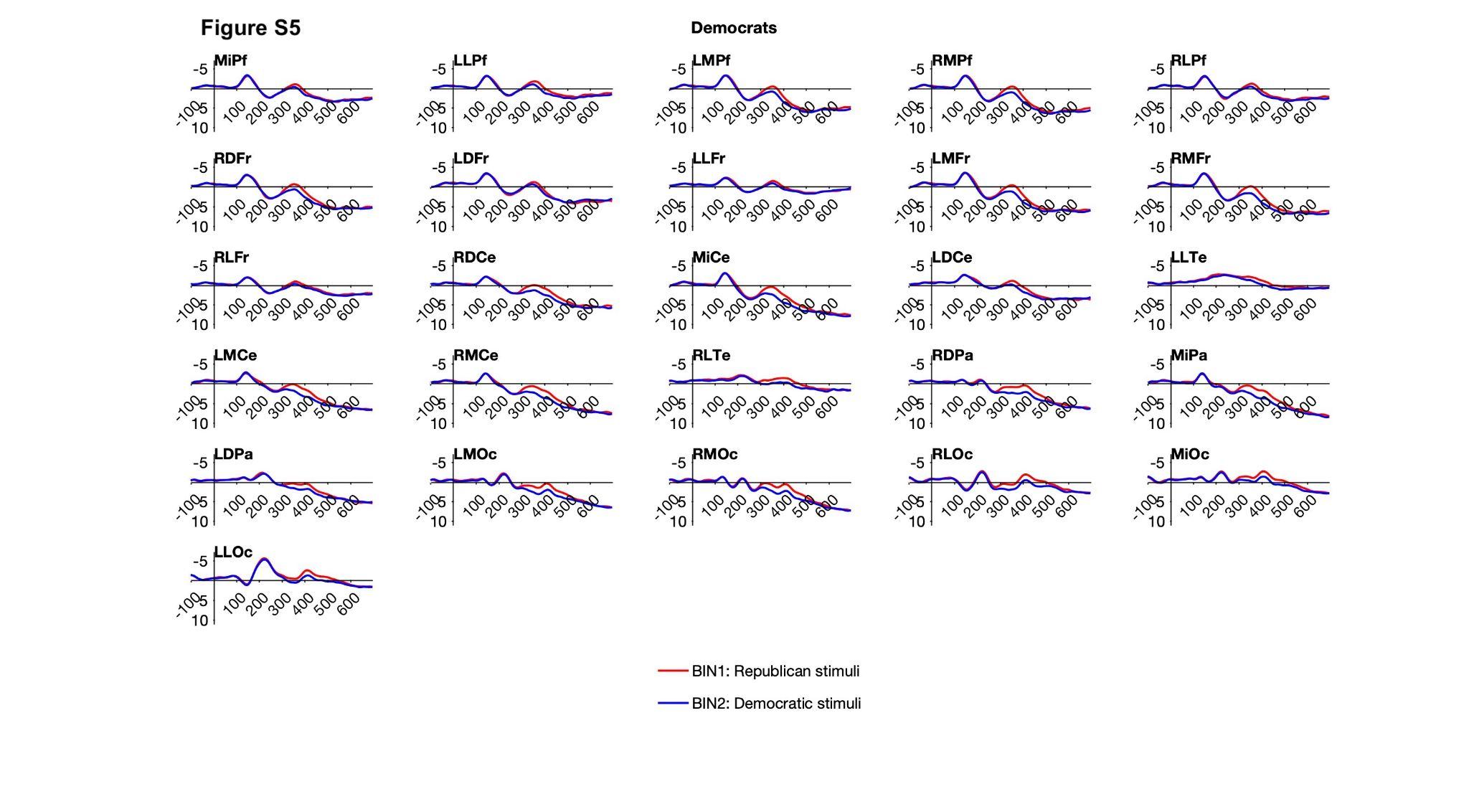
**

**Figure S5** shows the grand averaged waveforms of decided Democratic participants. Democratic statements are in blue and Republican statements in red. Waveforms are shown for every electrode from -100ms to 600ms. Please refer to supplementary figure S9 for the electrode site location.

**
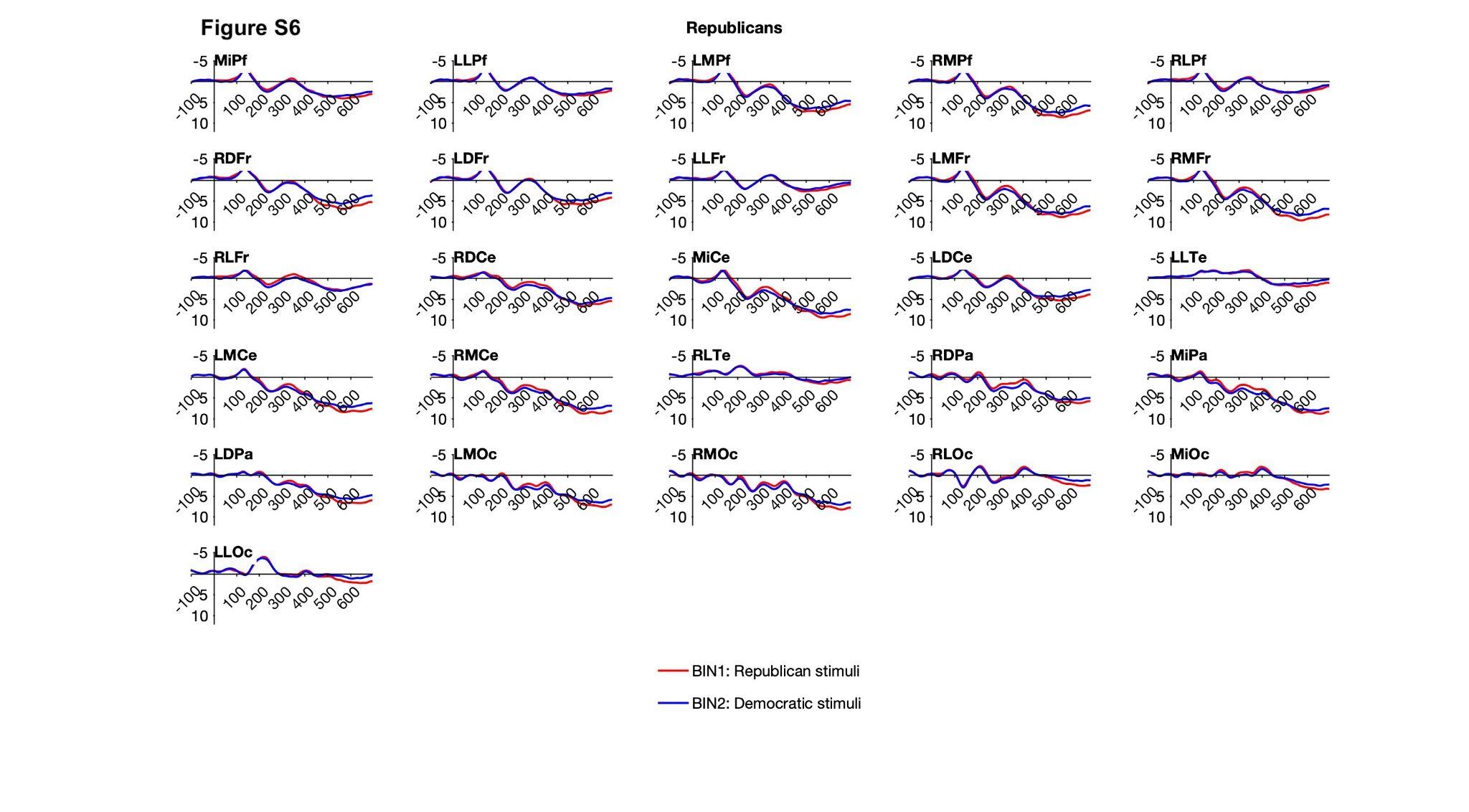
**

**Figure S6** shows the grand averaged waveforms of decided Republican participants. Democratic statements are in blue and Republican statements in red. Waveforms are shown for every electrode from -100ms to 600ms. Please refer to supplementary figure S9 for the electrode site location.

**
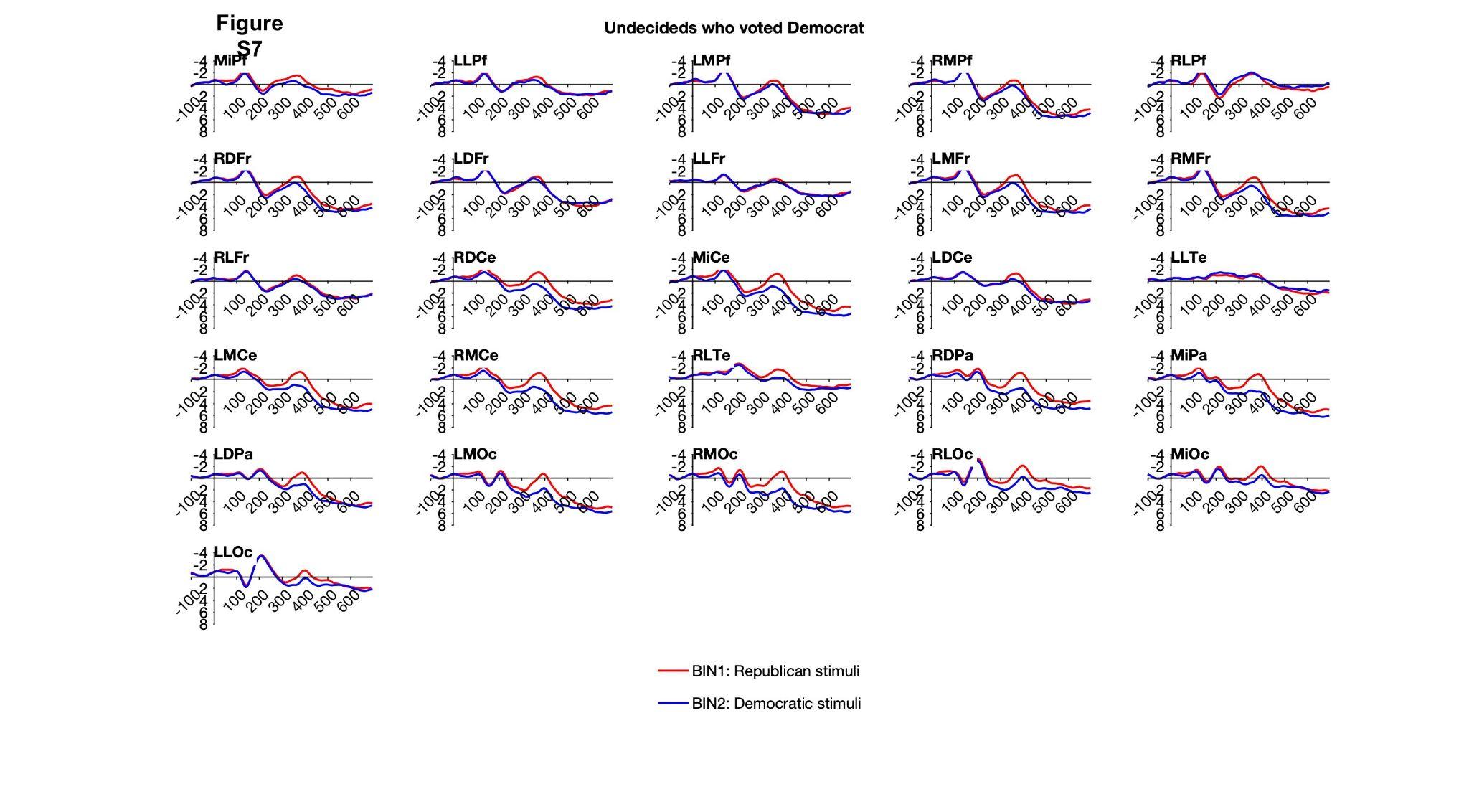
**

**Figure S7** shows the grand averaged waveforms of undecided participants who voted for the Democratic party. Democratic statements are in blue and Republican statements in red. Waveforms are shown for every electrode from -100ms to 600ms. Please refer to supplementary figure S9 for the electrode site location.

**
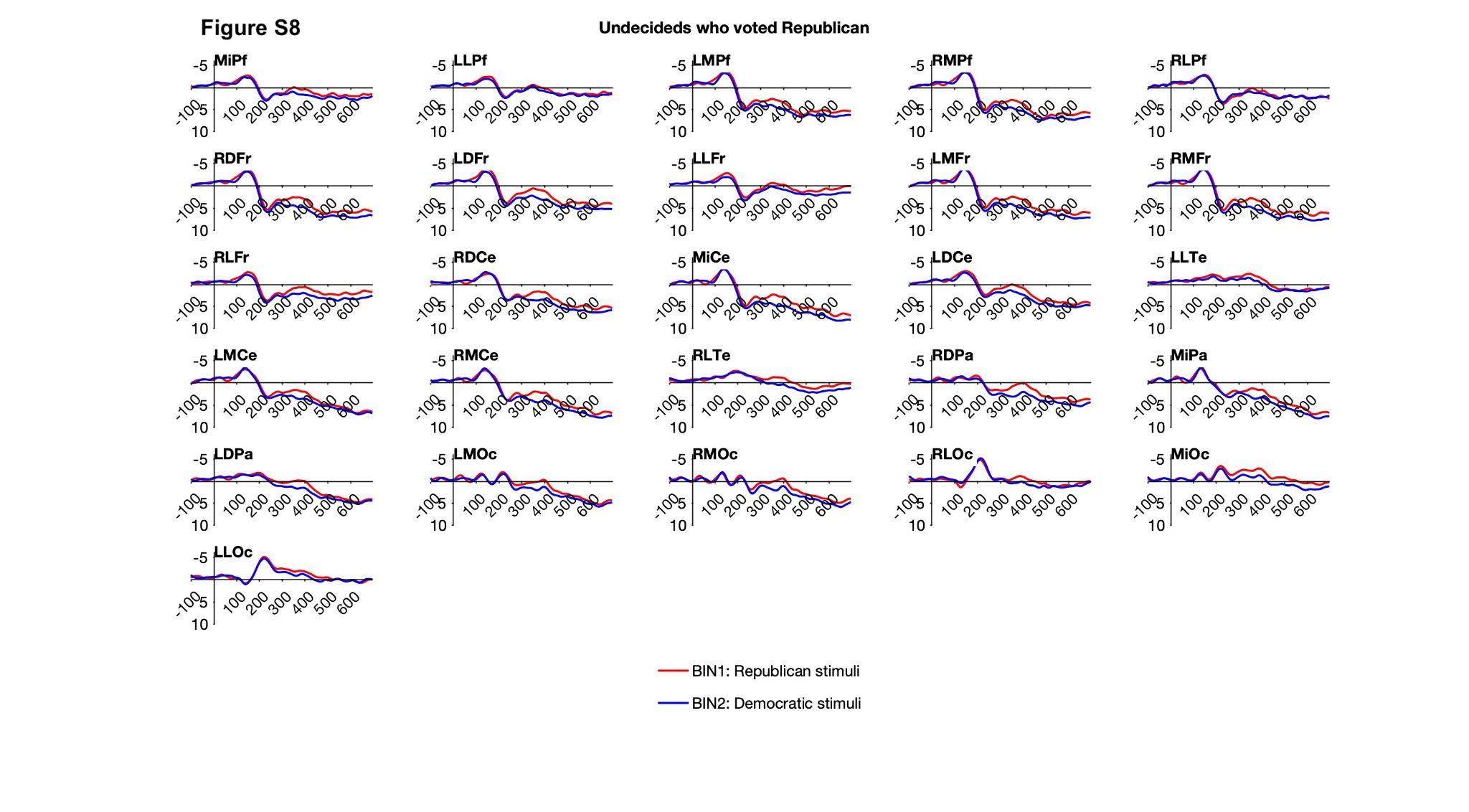
**

**Figure S8** shows the grand averaged waveforms of undecided participants who voted for the Republican party. Democratic statements are in blue and Republican statements in red. Waveforms are shown for every electrode from -100ms to 600ms. Please refer to supplementary figure S9 for the electrode site location.

**Figure S9**


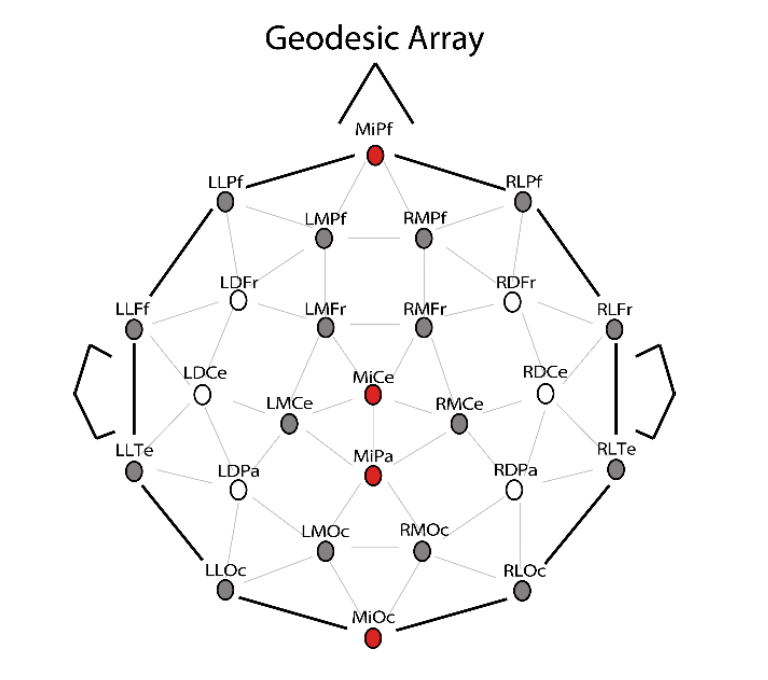


Figure S9 shows the Kutas electrode array used in this experiment. All 26 electrodes shown were used for the omnibus ANOVA. Electrodes in gray were used for the distributional analysis.

**Figure S10**

**Figure S10**. Individual participants are shown with their respective N400 effect values (in µV) for Economic issues and EPI scores for Economic issues. The black line shows the regression line when regressing the N400 effect on the EPI score.

**Figure S11**

**Figure S11**. Individual participants are shown with their respective N400 effect values (in µV) for Immigration issues and EPI scores for Immigration issues. The black line shows the regression line when regressing the N400 effect on the EPI score.

**Figure S12**

**Figure S12**. Individual participants are shown with their respective N400 effect values (in µV) for Societal issues and EPI scores for Societal issues. The black line shows the regression line when regressing the N400 effect on the EPI score.

**Table S13**. Mean amplitude: Electrode by Statement Type

|  | Pre-frontal | | | | | Frontal | | | | | | Temporal | |
| --- | --- | --- | --- | --- | --- | --- | --- | --- | --- | --- | --- | --- | --- |
| Electrode | LLPf | LMPf | RMPf | RLPf | MIPf | LDFr | LMFr | RMFr | RDFr | LLFr | RLFr | LLTe | RLTe |
| Democratic statements | 0.99 | 3.52 | 3.91 | 0.95 | 1.54 | 2.31 | 4.22 | 4.48 | 3.15 | 0.96 | 1.38 | -0.05 | 0.49 |
| Republican statements | 0.58 | 2.83 | 3.16 | 0.81 | 1.06 | 1.94 | 3.14 | 3.44 | 2.34 | 0.76 | 0.86 | -0.39 | -0.23 |

|  | Central | | | | | Parietal | | | Occipital | | | | |
| --- | --- | --- | --- | --- | --- | --- | --- | --- | --- | --- | --- | --- | --- |
| Electrode | LDCe | LMCe | MICe | RMCe | RDCe | LDPa | MIPa | RDPa | LLOc | LMOc | RMOc | RLOc | MIOc |
| Democratic statements | 1.97 | 3.73 | 4.39 | 4.03 | 2.96 | 2.58 | 4.03 | 3.15 | 0.56 | 3.17 | 3.07 | 1.00 | 0.07 |
| Republican statements | 1.33 | 2.62 | 3.05 | 2.75 | 1.84 | 1.70 | 2.56 | 1.81 | -0.15 | 1.96 | 1.74 | -0.05 | -0.87 |

**Table S14**. Mean amplitude N400 effect (Republican minus Democratic statements) for all statements (Overall N400) and for Economy statements alone (in µV). Note that two participants had insufficient trials for the Economy issue dimension and were excluded.

|  | Overall N400 | | | N400 Economy | | |
| --- | --- | --- | --- | --- | --- | --- |
|  | Mean | Range | N | Mean | Range | N |
| Democratic voters | -1.6 | -4.49, 2.18 | 20 | -0.65 | -6.23, 4.71 | 19 |
| Republican voters | -0.76 | -2.81, 3.07 | 18 | 0.20 | -4.39, 6.50 | 15 |
| Undecided voters | -1.79 | -4.35, 0.92 | 17 | ​​-0.30 | -4.48, 5.26 | 16 |

**S15: Additional information on the five participants excluded from Issue Dimension-specific analyses**

We include the respective numbers of valid trials by statement type and issue dimension for the five participants who did not meet the threshold of 14 valid trials and were excluded from issue dimension-specific analyses:

First participant excluded:

Republican Economy Statements, Trial Count: 13

Democratic Economy Statements, Trial Count: 12

Republican Immigration Statements, Trial Count: 10

Democratic Immigration Statements, Trial Count: 13

Republican Societal Statements , Trial Count: 19

Democratic Societal Statements , Trial Count: 16

Second participant excluded:

Republican Economy Statements, Trial Count: 11

Democratic Economy Statements, Trial Count: 13

Republican Immigration Statements, Trial Count: 11

Democratic Immigration Statements, Trial Count: 11

Republican Societal Statements , Trial Count: 9

Democratic Societal Statements , Trial Count: 16

Third participant excluded:

Republican Economy Statements, Trial Count: 20

Democratic Economy Statements, Trial Count: 21

Republican Immigration Statements, Trial Count: 11

Democratic Immigration Statements, Trial Count: 15

Republican Societal Statements , Trial Count: 23

Democratic Societal Statements , Trial Count: 24

Fourth participant excluded:

Republican Economy Statements, Trial Count: 16

Democratic Economy Statements, Trial Count: 7

Republican Immigration Statements, Trial Count: 8

Democratic Immigration Statements, Trial Count: 11

Republican Societal Statements , Trial Count: 12

Democratic Societal Statements , Trial Count: 11

Fifth participant excluded:

Republican Economy Statements, Trial Count: 10

Democratic Economy Statements, Trial Count: 9

Republican Immigration Statements, Trial Count: 8

Democratic Immigration Statements, Trial Count: 8

Republican Societal Statements , Trial Count: 13

Democratic Societal Statements , Trial Count: 18

**S16: Statements**

**Practice trials**

I think the second amendment of the Constitution should be changed.

Social distancing measures to avoid catching covid should now be stopped.

The governor's answer to rising crime should be spending on education.

Environmental legislation is a way for the government to introduce taxes.

Allowing people to carry guns without a background check is dangerous.

I think that politicians who support sanctuary cities should be prosecuted.

The price of certain prescription drugs like insulin should be capped.

What Texas workers need to uphold their dignity is fewer taxes.

To those who receive them vaccines against covid-19 offer considerable protection.

The main beneficiary of the Inflation Reduction Package is the IRS.

**Block A**

On the matter of drugs I want marijuana to be criminalized.

In terms of the services it provides government should be bigger.

We see an immigration crisis because of the policies of Biden.

The profit made by business corporations in the US is excessive.

I think that a government run healthcare system would limit inequalities.

Speaking of the distribution of wealth America should have fewer socialists.

Soaring prices and inflation will be dealt with by a Republican.

In their contribution to American culture immigrants bring American values strength.

The people who stormed the Capitol on January 6th were patriots.

In my opinion, the Texas State abortion ban should be maintained.

Taxpayer money spent on policing the border with Mexico is wasted.

To prevent killings in Texas we should have more gun sales.

The Justice Department's inquiry into Donald Trump's Florida home is warranted.

President Joe Biden's economic policies have led to increases in prices.

I think raising the minimum wage would lead to job losses.

It's my belief that man made climate change is a threat.

The Biden administration has pursued economic policies that resulted in inflation.

The government should make birth control pills for women more available.

Governor Abbott's management of the covid-19 pandemic has been a success.

When they try to enter the US immigrants should be welcomed.

The way governor Abbott deals with gun violence has my approval.

When responding to the pandemic, stay at home orders were right.

Tax rates on the highest incomes in America should be raised.

The state governor should treat unauthorized immigrants in Texas as felons.

For energy generation, Texas should invest a lot more in renewables.

Sending illegal immigrants from Texas to Washington by bus is right.

The candidate who will keep Texas rural hospitals open is Abbott.

For US society a greater acceptance of transgender people is moral.

In the economic policies he pursued, governor Abbott has driven growth.

The real culprit of the Southern border crisis is the governor.

The increased public focus on the history of slavery is welcome.

My own views on the Black Lives Matter movement are negative.

I think the governor who will support law enforcement is Beto.

Free public healthcare for Texan families and individuals is a right.

I think that gun control laws and regulations should be loosened.

The committee investigating the January 6th incident is pursuing the truth.

Over the years the energy industry in Texas has brought pollution.

I think that emergency measures to combat illegal immigration are necessary.

Building a wall on the entire border with Mexico is wrong.

The labor shortages in this country could be filled by Americans.

Security and public order is an issue best handled by Republicans.

The Democrats' economic agenda will lead the US economy to growth.

On abortion, the most important rights are those of the woman.

The Black Lives Matter protesters in 2020 were asking for trouble.

The ownership of assault weapons like AR-15s needs to be restricted.

Across the country Obamacare has made American families and individuals poorer.

I think affirmative action programmes for African Americans should be expanded.

We are all much better off with a president like Trump.

I personally think that sanctuary cities in Texas should be allowed.

Building a wall at the border would make US more secure.

People who say that America has racist institutions have a point.

Given how it handled BLM protests the police should be applauded.

Prices are rising and will keep doing so because of Abbott.

The decision to interrupt a pregnancy is something up to God.

When he left office, Donald Trump handled sensitive classified information appropriately.

In my opinion the country's economy is best handled by Democrats.

Speaking about abortion, we need a governor who is pro- life.

Expanding Medicaid in Texas would make our state healthcare system better.

I expect my representatives to confirm that abortion is a right.

Opening America to people all over the world makes it weaker.

Under the Affordable Care Act, the quality of healthcare has improved.

I think imposing a minimum tax on large corporations is unfair.

The covid restrictions that were imposed in the US were unnecessary.

Criminalising all abortions even in the case of rape is right.

The fact that some people in America are billionaires is unfair.

Women crossing state lines to have an abortion should be punished.

Under Joe Biden the price of gas has become considerably controlled.

Immigrants contribute to the Texas economy in a way that's negative.

People who stormed the Capitol on January 6th should be thanked.

The ban on abortions in Texas is something I find appalling.

Forgiving student loans like President Joe Biden recently did is socialist.

Proof of vaccination against covid-19 for public employees should be mandatory.

Federal and state funding for planned parenthood should be significantly increased.

When it interacts with African American citizens, the police is fair.

The people who want to restrict gun rights are against violence.

Under Obamacare the cost of health insurance for ordinary people increased.

Creating a social safety net for the most vulnerable is socialist.

Letting people come across the Southern border with Mexico is humane.

I strongly believe that the 2020 presidential election results were fair.

I think spending public money to welcome asylum seekers is wrong.

If they meet certain requirements illegal immigrants already here should stay.

For Texans what a Republican governor would mean is more unemployment.

Governor Greg Abbott's border operation Lone Star is a total success.

The Republicans' economic agenda will lead the US economy to recession.

By investigating Donald Trump, the Justice Department is doing its witchhunt.

I think that affirmative action programs for African Americans are fair.

I think that accepting LGBTQ people in our communities is wrong.

Red flag laws on gun purchases and background checks are needed.

When it overturned Roe versus Wade the Supreme Court was right.

The death of George Floyd in 2020 was caused by accident.

The fact that Trump took classified information with him is outrageous.

I think the best protection we have against covid-19 is freedom.

**Block B**

Letting people come across the Southern border with Mexico is dangerous.

When responding to the pandemic, stay at home orders were wrong.

I think raising the minimum wage would lead to job gains.

When they try to enter the US immigrants should be stopped.

Women crossing state lines to have an abortion should be helped.

The fact that some people in America are billionaires is good.

It's my belief that man made climate change is a hoax.

In my opinion, the Texas State abortion ban should be repealed.

I think that accepting LGBTQ people in our communities is right.

In terms of the services it provides government should be smaller.

When it interacts with African American citizens, the police is biased.

Building a wall at the border would make US more isolated.

The Republicans' economic agenda will lead the US economy to growth.

I think imposing a minimum tax on large corporations is fair.

I think spending public money to welcome asylum seekers is right.

In their contribution to American culture immigrants bring American values harm.

I think that emergency measures to combat illegal immigration are unnecessary.

I think that a government run healthcare system would limit choices.

To prevent killings in Texas we should have more gun restrictions.

The Democrats' economic agenda will lead the US economy to recession.

My own views on the Black Lives Matter movement are positive.

I strongly believe that the 2020 presidential election results were rigged.

Forgiving student loans like President Joe Biden recently did is right.

The increased public focus on the history of slavery is unwelcome.

The Biden administration has pursued economic policies that resulted in jobs.

Expanding Medicaid in Texas would make our state healthcare system worse.

The death of George Floyd in 2020 was caused by hatred.

For Texans what a Democratic governor would mean is more unemployment.

Speaking of the distribution of wealth America should have fewer billionaires.

For energy generation, Texas should invest a lot more in gas.

When it overturned Roe versus Wade the Supreme Court was wrong.

Taxpayer money spent on policing the border with Mexico is needed.

On abortion, the most important rights are those of the baby.

When he left office, Donald Trump handled sensitive classified information inappropriately.

Red flag laws on gun purchases and background checks are unconstitutional.

Building a wall on the entire border with Mexico is wise.

Criminalising all abortions even in the case of rape is outrageous.

The ban on abortions in Texas is something I find good.

Federal and state funding for planned parenthood should be significantly reduced.

The covid restrictions that were imposed in the US were necessary.

Tax rates on the highest incomes in America should be lowered.

President Joe Biden's economic policies have led to increases in growth.

Opening America to people all over the world makes it stronger.

The people who stormed the Capitol on January 6th were thugs.

The Justice Department's inquiry into Donald Trump's Florida home is unfair.

Proof of vaccination against covid-19 for public employees should be optional.

Governor Greg Abbott's border operation Lone Star is a total failure.

The Black Lives Matter protesters in 2020 were asking for justice.

I personally think that sanctuary cities in Texas should be banned.

The profit made by business corporations in the US is fair.

Over the years the energy industry in Texas has brought prosperity.

Sending illegal immigrants from Texas to Washington by bus is wrong.

By investigating Donald Trump, the Justice Department is doing its duty.

The ownership of assault weapons like AR-15s needs to be protected.

We see an immigration crisis because of the policies of Abbott.

I think that gun control laws and regulations should be tightened.

Under the Affordable Care Act, the quality of healthcare has worsened.

The labor shortages in this country could be filled by migrants.

The candidate who will keep Texas rural hospitals open is Beto.

I think that affirmative action programmes for African Americans are unfair.

Soaring prices and inflation will be dealt with by a Democrat.

The way governor Abbott deals with gun violence has my disapproval.

The fact that Trump took classified information with him is irrelevant.

The government should make birth control pills for women more unavailable.

Governor Abbott's management of the covid-19 pandemic has been a failure.

I think the governor who will support law enforcement is Abbott.

Creating a social safety net for the most vulnerable is important.

The state governor should treat unauthorized immigrants in Texas as refugees.

The committee investigating the January 6th incident is pursuing the witchhunt.

The people who want to restrict gun rights are against freedom.

Under Obamacare the cost of health insurance for ordinary people decreased.

In my opinion the country's economy is best handled by Republicans.

For US society a greater acceptance of transgender people is immoral.

Immigrants contribute to the Texas economy in a way that's positive.

If they meet certain requirements illegal immigrants already here should leave.

I think the best protection we have against covid-19 is vaccines.

Security and public order is an issue best handled by Democrats.

Under Joe Biden the price of gas has become considerably costlier.

I expect my representatives to confirm that abortion is a crime.

We are all much better off with a president like Biden.

Across the country Obamacare has made American families and individuals healthier.

I think affirmative action programs for African Americans should be abolished.

Given how it handled BLM protests the police should be defunded.

The real culprit of the Southern border crisis is the president.

On the matter of drugs I want marijuana to be legalized.

Speaking about abortion, we need a governor who is pro- choice.

People who say that America has racist institutions have a problem.

In the economic policies he pursued, governor Abbott has driven inflation.

Free public healthcare for Texan families and individuals is a trap.

The decision to interrupt a pregnancy is something up to women.

People who stormed the Capitol on January 6th should be prosecuted.

Prices are rising and will keep doing so because of Biden.

#### S17: Stimuli not covered by the Warriner et al. valence database

**word statement type Valence**

inequality pro-Democratic Negative

defund pro-Democratic Negative

renewable pro-Democratic Positive

repeal pro-Democratic Positive

legalise pro-Democratic Positive

American pro-Republican Positive

witchhunt pro-Republican Negative

witchhunt pro-Republican Negative

unconstitutional pro-Republican Negative

lower pro-Republican Negative

worsen pro-Republican Negative

#### Table S18. Stimuli valence

|  | Database  word | Experimental  stimulus | Statement  type | Trial  codes | Valence  score |
| --- | --- | --- | --- | --- | --- |
| 1 | abolish | abolished | pro-Republican | 138 | 3,84 |
| 2 | accident | accident | pro-Republican | 114 | 2,55 |
| 3 | allow | allowed | pro-Democrat | 60 | 6,39 |
| 4 | appalling | appalling | pro-Democrat | 106 | 2,95 |
| 5 | applaud | applauded | pro-Republican | 100 | 6,7 |
| 6 | approval | approval | pro-Republican | 90 | 6,75 |
| 7 | appropriate | appropriately | pro-Republican | 173 | 6,1 |
| 8 | available | available | pro-Democrat | 89 | 6,86 |
| 9 | baby | baby | pro-Republican | 124 | 6,67 |
| 10 | bad | worse | pro-Republican | 158 | 3,24 |
| 11 | biased | biased | pro-Democrat | 118 | 4,21 |
| 12 | billionaire | billionaires | pro-Democrat | 37 | 6 |
| 13 | choice | choice | pro-Democrat | 141 | 6,36 |
| 14 | choice | choices | pro-Republican | 157 | 6,36 |
| 15 | control | controlled | pro-Democrat | 22 | 4,43 |
| 16 | costly | costlier | pro-Republican | 46 | 3 |
| 17 | criminal | criminalized | pro-Republican | 86 | 2,11 |
| 18 | crime | crime | pro-Republican | 137 | 1,95 |
| 19 | dangerous | dangerous | pro-Republican | 68 | 2,33 |
| 20 | decrease | decreased | pro-Democrat | 164 | 4,16 |
| 21 | disapproval | disapproval | pro-Democrat | 133 | 3,4 |
| 22 | duty | duty | pro-Democrat | 182 | 5,33 |
| 23 | excessive | excessive | pro-Democrat | 5 | 4,37 |
| 24 | expand | expanded | pro-Democrat | 98 | 5,35 |
| 25 | failure | failure | pro-Democrat | 163 | 2,15 |
| 26 | failure | failure | pro-Democrat | 77 | 2,15 |
| 27 | fair | fair | pro-Democrat | 110 | 7,14 |
| 28 | fair | fair | pro-Republican | 41 | 7,14 |
| 29 | fair | fair | pro-Republican | 108 | 7,14 |
| 30 | fair | fair | pro-Democrat | 32 | 7,14 |
| 31 | fair | fair | pro-Democrat | 175 | 7,14 |
| 32 | felon | felons | pro-Republican | 54 | 2,2 |
| 33 | freedom | freedom | pro-Republican | 155 | 7,72 |
| 34 | freedom | freedom | pro-Republican | 135 | 7,72 |
| 35 | gain | gains | pro-Democrat | 27 | 5,9 |
| 36 | gas | gas | pro-Republican | 38 | 4,06 |
| 37 | good | good | pro-Republican | 127 | 7,89 |
| 38 | good | good | pro-Republican | 28 | 7,89 |
| 39 | good | better | pro-Democrat | 150 | 7,89 |
| 40 | governor | governor | pro-Democrat | 56 | 5,32 |
| 41 | growth | growth | pro-Democrat | 40 | 6 |
| 42 | growth | growth | pro-Republican | 31 | 6 |
| 43 | growth | growth | pro-Democrat | 16 | 6 |
| 44 | growth | growth | pro-Republican | 14 | 6 |
| 45 | harm | harm | pro-Republican | 72 | 1,91 |
| 46 | hatred | hatred | pro-Democrat | 122 | 2,38 |
| 47 | healthy | healthier | pro-Democrat | 166 | 7,76 |
| 48 | help | helped | pro-Democrat | 115 | 6,95 |
| 49 | hoax | hoax | pro-Republican | 29 | 3,85 |
| 50 | humane | humane | pro-Democrat | 64 | 6,88 |
| 51 | immoral | immoral | pro-Republican | 136 | 2,79 |
| 52 | important | important | pro-Democrat | 44 | 6,82 |
| 53 | improve | improved | pro-Democrat | 151 | 6,14 |
| 54 | inappropriate | inappropriately | pro-Democrat | 179 | 3,7 |
| 55 | increase | increased | pro-Republican | 154 | 5,9 |
| 56 | increase | increased | pro-Democrat | 107 | 5,9 |
| 57 | inflation | inflation | pro-Republican | 11 | 2,91 |
| 58 | inflation | inflation | pro-Democrat | 48 | 2,91 |
| 59 | irrelevant | irrelevant | pro-Republican | 183 | 3,35 |
| 60 | isolate | isolated | pro-Democrat | 70 | 4,35 |
| 61 | justice | justice | pro-Democrat | 129 | 6,8 |
| 62 | leave | leave | pro-Republican | 84 | 4,68 |
| 63 | life | life | pro-Republican | 102 | 6,68 |
| 64 | loosen | loosened | pro-Republican | 94 | 5 |
| 65 | loss | losses | pro-Republican | 9 | 2,9 |
| 66 | maintain | maintained | pro-Republican | 87 | 6,29 |
| 67 | mandatory | mandatory | pro-Democrat | 153 | 3,9 |
| 68 | moral | moral | pro-Democrat | 91 | 6,85 |
| 69 | necessary | necessary | pro-Republican | 57 | 5,39 |
| 70 | necessary | necessary | pro-Democrat | 159 | 5,39 |
| 71 | need | needed | pro-Republican | 74 | 5,45 |
| 72 | need | needed | pro-Democrat | 112 | 5,45 |
| 73 | negative | negative | pro-Republican | 93 | 2,52 |
| 74 | negative | negative | pro-Republican | 63 | 2,52 |
| 75 | optional | optional | pro-Republican | 160 | 5,76 |
| 76 | outrageous | outrageous | pro-Democrat | 177 | 4,19 |
| 77 | outrageous | outrageous | pro-Democrat | 126 | 4,19 |
| 78 | patriot | patriots | pro-Republican | 168 | 5,8 |
| 79 | point | point | pro-Democrat | 99 | 5,45 |
| 80 | pollution | pollution | pro-Democrat | 15 | 2 |
| 81 | poor | poorer | pro-Republican | 149 | 3,67 |
| 82 | positive | positive | pro-Democrat | 83 | 7,57 |
| 83 | positive | positive | pro-Democrat | 120 | 7,57 |
| 84 | president | president | pro-Republican | 85 | 5,23 |
| 85 | price | prices | pro-Republican | 8 | 4,94 |
| 86 | problem | problem | pro-Republican | 142 | 3,52 |
| 87 | prosperity | prosperity | pro-Republican | 42 | 7,1 |
| 88 | prosecute | prosecuted | pro-Democrat | 187 | 3,15 |
| 89 | protected | protected | pro-Republican | 130 | 7,05 |
| 90 | punish | punished | pro-Republican | 105 | 2,86 |
| 91 | raise | raised | pro-Democrat | 12 | 7,22 |
| 92 | recession | recession | pro-Democrat | 26 | 2,68 |
| 93 | recession | recession | pro-Republican | 33 | 2,68 |
| 94 | refugee | refugees | pro-Democrat | 82 | 4,68 |
| 95 | restricted | restricted | pro-Democrat | 97 | 3,83 |
| 96 | restriction | restrictions | pro-Democrat | 119 | 4 |
| 97 | right | right | pro-Republican | 113 | 7,32 |
| 98 | right | right | pro-Democrat | 34 | 7,32 |
| 99 | right | right | pro-Democrat | 117 | 7,32 |
| 100 | right | right | pro-Republican | 55 | 7,32 |
| 101 | right | right | pro-Democrat | 146 | 7,32 |
| 102 | right | right | pro-Republican | 104 | 7,32 |
| 103 | right | right | pro-Democrat | 103 | 7,32 |
| 104 | right | right | pro-Democrat | 71 | 7,32 |
| 105 | right | right | pro-Democrat | 148 | 7,32 |
| 106 | sale | sales | pro-Republican | 88 | 6,23 |
| 107 | secure | secure | pro-Republican | 61 | 7,08 |
| 108 | small | smaller | pro-Republican | 30 | 5,76 |
| 109 | socialist | socialist | pro-Republican | 23 | 4,11 |
| 110 | socialist | socialist | pro-Republican | 24 | 4,11 |
| 111 | socialist | socialists | pro-Republican | 6 | 4,11 |
| 112 | stay | stay | pro-Democrat | 66 | 6,14 |
| 113 | stop | stopped | pro-Republican | 69 | 4,73 |
| 114 | strength | strength | pro-Democrat | 51 | 6,73 |
| 115 | strong | stronger | pro-Democrat | 76 | 6,81 |
| 116 | success | success | pro-Republican | 145 | 7,49 |
| 117 | success | success | pro-Republican | 67 | 7,49 |
| 118 | thank | thanked | pro-Republican | 174 | 7,77 |
| 119 | threat | threat | pro-Democrat | 10 | 2,63 |
| 120 | thug | thugs | pro-Democrat | 180 | 2,52 |
| 121 | tighten | tightened | pro-Democrat | 131 | 5 |
| 122 | trap | trap | pro-Republican | 167 | 3,24 |
| 123 | trouble | trouble | pro-Republican | 96 | 2,87 |
| 124 | truth | truth | pro-Democrat | 171 | 7,19 |
| 125 | unavailable | unavailable | pro-Republican | 134 | 3,52 |
| 126 | unemployment | unemployment | pro-Democrat | 25 | 2,32 |
| 127 | unemployment | unemployment | pro-Republican | 36 | 2,32 |
| 128 | unfair | unfair | pro-Democrat | 21 | 3,15 |
| 129 | unfair | unfair | pro-Republican | 132 | 3,15 |
| 130 | unfair | unfair | pro-Republican | 181 | 3,15 |
| 131 | unfair | unfair | pro-Republican | 20 | 3,15 |
| 132 | unnecessary | unnecessary | pro-Democrat | 73 | 4,62 |
| 133 | unnecessary | unnecessary | pro-Republican | 152 | 4,62 |
| 134 | unwelcome | unwelcome | pro-Republican | 121 | 3,5 |
| 135 | vaccine | vaccines | pro-Democrat | 165 | 6,48 |
| 136 | violence | violence | pro-Democrat | 109 | 2,71 |
| 137 | warrant | warranted | pro-Democrat | 169 | 3,23 |
| 138 | waste | wasted | pro-Democrat | 52 | 2,61 |
| 139 | weak | weaker | pro-Republican | 62 | 2,95 |
| 140 | welcome | welcomed | pro-Democrat | 53 | 7,27 |
| 141 | welcome | welcome | pro-Democrat | 92 | 7,27 |
| 142 | welcome | welcomed | pro-Democrat | 53 | 7,27 |
| 143 | welcome | welcome | pro-Democrat | 92 | 7,27 |
| 144 | wise | wise | pro-Republican | 75 | 7,42 |
| 145 | woman | woman | pro-Democrat | 95 | 7,09 |
| 146 | wrong | wrong | pro-Democrat | 79 | 3,24 |
| 147 | wrong | wrong | pro-Republican | 156 | 3,24 |
| 148 | wrong | wrong | pro-Republican | 65 | 3,24 |
| 149 | wrong | wrong | pro-Democrat | 58 | 3,24 |
| 150 | wrong | wrong | pro-Democrat | 123 | 3,24 |
| 151 | wrong | wrong | pro-Republican | 111 | 3,24 |
| 152 | ban | banned | pro-Republican | 78 | 3,38 |
| 153 | big | bigger | pro-Democrat | 4 | 5,64 |
| 154 | democrat | Democrat | pro-Democrat | 43 | 5,54 |
| 155 | democrat | Democrats | pro-Democrat | 19 | 5,54 |
| 156 | democrat | Democrats | pro-Democrat | 186 | 5,54 |
| 157 | god | God | pro-Republican | 101 | 5,9 |
| 158 | immigrant | migrants | pro-Democrat | 81 | 5,43 |
| 159 | job | jobs | pro-Democrat | 35 | 5,64 |
| 160 | republican | Republican | pro-Republican | 7 | 4,26 |
| 161 | republican | Republicans | pro-Republican | 172 | 4,26 |
| 162 | republican | Republicans | pro-Republican | 45 | 4,26 |
| 163 | rig | rigged | pro-Republican | 178 | 4,32 |
| 164 | woman | women | pro-Democrat | 143 | 7,09 |

**Figure S19.** EPI scores for Democratic, Republican and undecided voters across 3 issue dimensions
